# Proteogenomic characterization of cholangiocarcinoma

**DOI:** 10.1002/hep.32624

**Published:** 2022-07-05

**Authors:** Mengjie Deng, Peng Ran, Lingli Chen, Yunzhi Wang, Zixiang Yu, Ke Cai, Jinwen Feng, Zhaoyu Qin, Yanan Yin, Subei Tan, Yang Liu, Chen Xu, Guoming Shi, Yuan Ji, Jian‐Yuan Zhao, Jian Zhou, Jia Fan, Yingyong Hou, Chen Ding

**Affiliations:** 1 State Key Laboratory of Genetic Engineering and Collaborative Innovation Center for Genetics and Development, School of Life Sciences, Institute of Biomedical Sciences, Human Phenome Institute, Zhongshan Hospital, Fudan University, Shanghai, China; 2 Department of Pathology, Zhongshan Hospital, Fudan University, Shanghai, China; 3 Department of Liver Surgery and Transplantation, Liver Cancer Institute, Zhongshan Hospital, Fudan University, Key Laboratory of Carcinogenesis and Cancer Invasion of Ministry of Education, Shanghai, China; 4 Institute for Development and Regenerative Cardiovascular Medicine, MOE‐Shanghai Key Laboratory of Children's Environmental Health, Xinhua Hospital, Shanghai Jiao Tong University School of Medicine, Shanghai, China; 5 Key Laboratory of Medical Epigenetics and Metabolism, Institutes of Biomedical Sciences, Fudan University, Shanghai, China

## Abstract

Cholangiocarcinoma (CCA) is a highly heterogeneous cancer with limited understanding and few effective therapeutic approaches. We aimed at providing a proteogenomic CCA characterization to inform biological processes and treatment vulnerabilities.

**Approach and Results:**

Integrative genomic analysis with functional validation uncovered biological perturbations downstream of driver events including *DPCR1*, *RBM47* mutations, *SH3BGRL2* copy number alterations, and *FGFR2* fusions in CCA. Proteomic clustering identified three subtypes with distinct clinical outcomes, molecular features, and potential therapeutics. Phosphoproteomics characterized targetable kinases in CCA, suggesting strategies for effective treatment with CDK and MAPK inhibitors. Patients with CCA with HBV infection showed increased antigen processing and presentation (APC) and T cell infiltration, conferring a favorable prognosis compared with those without HBV infection. The characterization of extrahepatic CCA recommended the feasible application of vascular endothelial‐derived growth factor inhibitors. Multiomics profiling presented distinctive molecular characteristics of the large bile duct and the small bile duct of intrahepatic CCA. The immune landscape further revealed diverse tumor immune microenvironments, suggesting immune subtypes C1 and C5 might benefit from immune checkpoint therapy. TCN1 was identified as a potential CCA prognostic biomarker, promoting cell growth by enhancing vitamin B12 metabolism.

**Conclusions:**

We characterized the proteogenomic landscape of 217 CCAs with 197 paired normal adjacent tissues and identified their subtypes and potential therapeutic targets. The multiomics analyses with other databases and some functional validations have indicated strategies regarding the clinical, biological, and therapeutic approaches to the management of CCA.

## Graphical abstract

**Figure:**
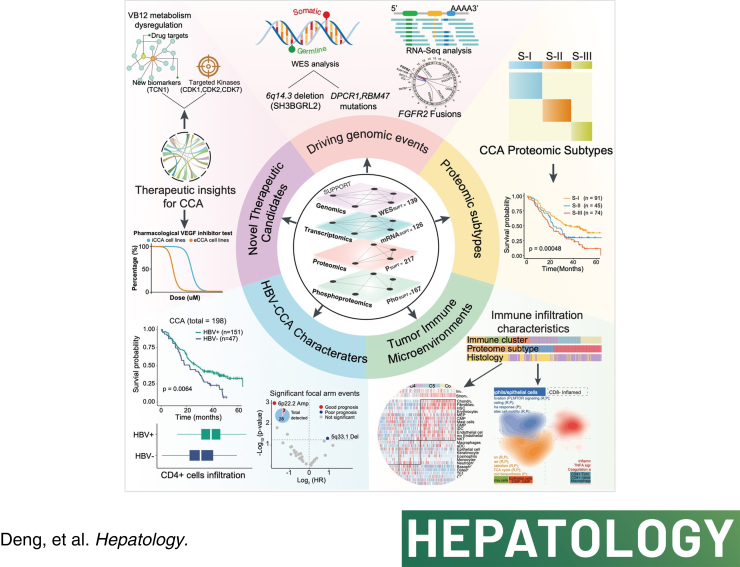

## INTRODUCTION

Cholangiocarcinoma (CCA) is the second most common primary liver cancer, with the 5‐year survival being approximately 5%–15%.^1^ CCAs are anatomically classified as intrahepatic and extrahepatic CCA (iCCA and eCCA). iCCA can be stratified into small bile duct iCCA (small iBD) and large bile duct iCCA (large iBD) based on the level or size of the affected ducts, exhibiting high molecular heterogeneity with different cells of origin and pathogenesis. HBV infections have been identified as CCA risk factors, yet its impacts on CCA and the mechanism underneath remain unclarified.^1^,^2^

Next‐generation sequencing‐based studies have uncovered the CCA genetic landscape,^3^ revealing the shared recurrent mutations in *TP53*, *ARID1A*, *KRAS*, *SMAD4*, etc. However, the complete understanding of genetic aberrations‐driven tumor phenotypes remains ambiguous. Despite treatments based on *IDH* mutations^4^ and *FGFR* fusions^5^ having preliminary clinical efficacy in iCCA, the knowledge of eCCA molecular variants is limited due to the deficiency of effective targeted therapies.

Recent immune checkpoint inhibitors (ICIs) targeting PD‐1 or CTLA‐4 have shown great potential for tumor‐specific immune disinhibitions, with durable efficacy and low immune‐related adverse event rates.^6^ However, reliance on genomic and transcriptomic findings alone for improved CCA therapy is limited and in need of additional research methods. Proteogenomic‐based tumor immune microenvironment analyses can provide insights into ICI selection criteria and immunotherapy targets.

Herein, we performed an integrated multiomic analysis of 217 CCAs and 197 normal adjacent tissues (NATs), providing a comprehensive report on the systematic CCA biological process.

## PATIENTS AND METHODS

### Patient cohort

We collected 217 cases (114 iCCAs and 103 eCCAs) with clinical and pathological records of patients with CCA who underwent primary curative resection from January 2012 to December 2018 at Zhongshan Hospital of Fudan University with the approval of the Research Ethics Committee (B2019‐200R). Patients with CCA on prior anticancer treatments were excluded from this study. The baseline characteristics of patients with CCA were summarized in Table S1.

### Sample preparation

Formalin‐fixed, paraffin‐embedded (FFPE) specimens were prepared and provided by Zhongshan Hospital. For genomic, proteomic, and phosphoproteomic sample preparation, 10 μm slides were deparaffinized with xylene and washed with gradient ethanol. For RNA sample preparation, 10 μm slides were prepared from the samples without xylene deparaffination or gradient ethanol wash. The specimens were selected according to H&E staining status and scraped.

### Multiomics data analysis

**FIGURE 1:**
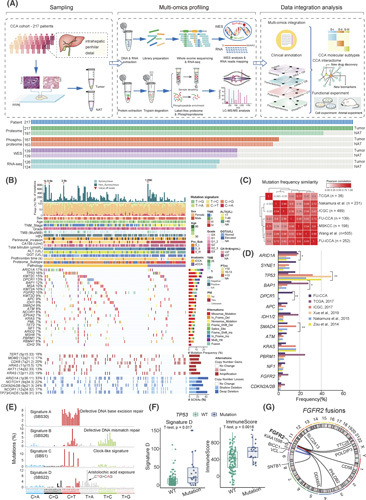
Multiomics landscape of cholangiocarcinoma (CCA). (A) Schematic representation of the experimental design (top panel), and heatmap representing the multiplatform data generated in this study. Gray blocks in the schematic represent the missing data. The annotations to the left indicate samples in each category (bottom panel). (B) Genomic landscape of CCA samples showing mutations in putative driver genes, including eight SMGs and 16 frequently, but not significantly, mutated genes (indicated with asterisks), ordered by their mutation frequencies in CCA. The bottom graph shows targeted genes by focal SCNAs: dark red for amplification, light red for gain, dark blue for deletion, and light blue for loss. The altered frequency of each gene is shown on the left with a bar chart on the right. (C) Correlation plot of the mutation frequencies observed in the FU‐CCA cohort compared to those in previously published cohorts (Spearman correlation). (D) Bar plots of mutational frequencies for genes between the FU‐CCA cohort and previously published CCA studies (Fisher's exact test). (E) Mutational spectrum of the four de novo mutational signatures. Corresponding to COSMIC signatures with similarities are labeled. (F) Boxplots showing the Signature D (SBS22) exposure and the immune score in *TP53*‐mutated and *TP53*‐WT groups (*t* test, *p* < 0.05). (G) Circos plot showing patients with CCA with *FGFR2* fusions identified by RNA‐seq data in the FU‐CCA cohort.

We performed whole‐exome sequencing (WES), RNA sequencing (RNA‐seq), proteome profiling, and phosphoproteome profiling (Figure 1A). Sample annotation, processed, and normalized data files are provided in Table S1. The detailed methods are presented in the Supporting Materials and Methods.

### Cell lines and treatments

Human CCA cell lines (iCCA: HCCC‐9810, HuCCT1, CCLP1, and TKKK; eCCA: QBC‐939, TFK‐1, and EGI‐1; cells were purchased from DSMZ and ATCC) were incubated in DMEM (high glucose) medium with 10% FBS, 100 U/ml penicillin, and 100 U/ml streptomycin in an incubator with 5% CO_2_ at 37°C.

### Immunohistochemistry

FFPE tissue sections of 10 μM thickness were stained in batches for detecting CD34, phosphorylation of CDK1‐T161, phosphorylation of MCM2‐S27, and phosphorylation of RB1‐T373. Immunohistochemistry (IHC) was performed using the Leica BOND‐MAX autostaining system, followed by detection with a Bond Polymer Refine Detection DS9800. Slides were imaged using an OLYMPUS BX43 microscope and processed using a Scanscope.

### Statistical methods

Standard statistical tests were used to analyze the multiomics data, including Fisher's exact test and Chi‐square test for categorical variables, Kruskal‐Wallis test and Student *t* test for statistically significant differences between subgroups of continuous variables, and Spearman and Pearson correlation for continuous variables versus continuous variables. Kaplan–Meier estimated overall survival with log‐rank tests among strata, and hazard ratio and confidence interval (CI) were calculated from Cox proportional hazards regression models. All statistical tests were two‐sided, and *p* value <0.05 was considered statistical significance. Full details and description of the statistical analysis methods are provided in the Supporting Materials and Methods.

## RESULTS

### Comprehensive proteogenomic characterization in CCA

We collected 217 primary CCA tumors and 197 NATs (Figure 1A; Figure S1A; Table S1). The clinical characteristics are summarized in Table S1. Comparison with published CCA datasets^3^,^7^–^10^ revealed the similarity of the patients' characteristics including age and sex, yet some distinctive features were also observed. At the racial and geographic level, our cohort, Zou et al.’s cohort, and the ICGC cohort were from Asia. Meanwhile, our cohort includes both patients with iCCA and those with eCCA, which enabled us to depict their molecular characteristics (Table S1). The FFPE samples were characterized by WES, RNA‐seq, and proteomic and phosphoproteomic profiling (Figure 1A).

WES led to 149‐fold mean target coverage and identified 12,572 mutated genes. RNA‐seq profiled 14,978 genes (12,263 protein‐coding genes) with FPKM >1 in 50% of samples. Proteomic analysis identified 14,994 proteins (Figure S1B,J; Table S1). Phosphoproteomic analysis identified 40,682 phosphopeptides, covering 32,219 highly reliable phosphosites (probability > 0.75) from 8398 phosphoproteins (Figure S1K).

The quality of the dataset was ensured through rigorous quality control (Figure S1C,F,G). The average correlation coefficients of the proteomic and phosphoproteomic standards were 0.94 (95% CI, 0.93–0.96) and 0.93 (95% CI, 0.90–0.97) (Figure S1D,E; Table S1). After appropriate filtering, we included 12,263 mRNAs, 6875 proteins, and 3398 phosphosites in the subsequent analysis (Table S1; Supporting Materials and Methods).

### Proteogenomic landscape and regulation in CCA

Eight significantly mutated genes (SMGs) were identified based on the WES, including *ARID1A, TP53*, *IDH1/2*, *DPCR1*, *BAP1*, *SMAD4*, *KRAS*, and *RBM47* (*q* < 0.1; Figure 1B; Figure S3A,B; Table S1; Supporting Material and Methods). The mutations of *ARID1A*, *TP53, BAP1*, *DPCR1*, and *SMAD4* were significantly different among different cohorts (Fisher's exact test adjusted *p* < 0.05; Figure 1C,D).^3^,^7^,^8^,^10^–^12^

Notably, the mutations of *RBM47* and *DPCR1* had not been reported in CCA. *RBM47* has been implicated as a tumor suppressor due to its role in regulating the P53/P21 pathway and cell cycle.^13^ *RBM47‐T42M* and *RBM47‐A284S* variants have been identified in functional domain regions based on AlphaFold Protein Structure Database (Figure S3C). Meanwhile, DPCR1, which encodes protein that modulates NFκb signaling pathway, has been reported to promote tumor cell proliferation in cancers.^14^ Consistently, *DPCR1* mutation significantly elevated its cognate protein expression (Figure S3G).

We conducted experiments to illustrate how the *RBM47* and *DPCR1* mutations influence the tumor progression of CCA. Four mutant variations of *RBM47* (*RBM47‐T42M*, *RBM47‐A284S*, *RBM47‐R405C*, and *RBM47‐426‐433del*) were generated and transfected into HCCC‐9810 and QBC‐939 cell lines. Comparing the cell lines transfected with the *RBM47*‐WT gene, those transfected with *RBM47* mutations demonstrated significantly higher cell proliferation rates (Figure S3D). Correspondingly, we found the protein expression of P53 was elevated in the *RBM47*‐mutant cell lines (Figure S3E,F), which suggested that *RBM47* mutations promoted CCA tumor cell proliferation by enhancing the P53/P21 pathway. Furthermore, we constructed four *DPCR1* mutation vectors (*DPCR1‐I949delinsTPL*, *DPCR1‐T907fs*, *DPCR1‐587_589del*, and *DPCR1‐S319P*) and transfected them into HCCC‐9810 and QBC‐939 cell lines. We found that cells transfected with *DCPR1* mutations significantly increased cell proliferation compared to cell lines that were transfected with *DCPR1*‐WT (Figure S3H). Meanwhile, we examined the impacts of *DPCR1* mutations on proteins that enriched in the NFκb signaling pathway and demonstrated that *DPCR1* mutations significantly increased the NFKBIA phosphorylation at Ser536 (Figure S3I,J). In general, these results confirmed the potential roles of *RBM47* and *DPCR1* mutations in promoting CCA proliferation.

WES analysis identified four mutational signatures^15^: A (SBS30), B (SBS26), C (SBS1), and D (SBS22; Figure 1E; Table S1). In total, 75.2% of patients with CCA were identified to have signature D of aristolochic acid (AA) exposure, which has been reported in iCCA and HCC in Chinese cohorts.^16^,^17^ We observed that patients with iCCA have more AA exposure than patients with eCCA (Figure S1H), and patients with AA exposure showed significantly higher *TP53* mutations and immune scores (Figure 1F; Figure S1I), Consistently, AA exposure has been reported to induce *TP53* mutation.^17^ These findings implicated that AA exposure had a critical role in CCA development.

### Proteogenomic characteristics associated with *FGFR2* alterations

*FGFR2* alterations are known oncogenic drivers in CCA. We performed gene fusion analysis and found nine patients with iCCA (10.8%) harbored *FGFR2* fusions (Figure 1G; Figure S4A; Supporting Materials and Methods). No *FGFR2* fusion events were observed in NAT or eCCA tumor samples.

Focusing on the fusion patterns of *FGFR2*, we conducted a comparative analysis with the recently published research.^11^ The fusion partners of *FGFR2*, including *KIAA1598*, *BICC1*, *VCL*, and *PHLDB2*, were detected in both studies. Six *FGFR2* fusion partners (*CD58*, *DDX60L*, *POLDIP3*, *SLC2A13*, *SNTB1*, and *TTC28*) were exclusively identified in our cohort (Figure S4B,C). In addition to *FGFR2*, other targetable gene fusions have also been identified (*TYK2*, *PDGFRA*, *DUSP22*, *KMT2A*, etc.; Table S1).

Six patients with iCCA were detected with *FGFR2* mutations (Figures S4D and S5A). We observed that *FGFR2* alteration (somatic mutations and gene fusions) exhibited significant *cis*‐effects on their cognate mRNAs and proteins (Figures S4E and S5B,C). We evaluated the ligands of FGFR2 and found no difference between *FGFR2* alteration and *FGFR2*‐WT groups (Figure S5D), suggesting that *FGFR2* alterations enabled self‐activation primarily through the increased expression of the corresponding FGFR2 protein. The *FGFR2* alterations also upregulated mRNAs/proteins involved in mRNA processing/splicing, PI3K‐Akt pathway, and cell cycle process (Figure S4F,G) as well as phosphoproteins enriched in Rho‐GTPase and Notch pathways (Figure S4G,H).

### Integrated proteogenomic analyses of genomic alterations in CCA

**FIGURE 2:**
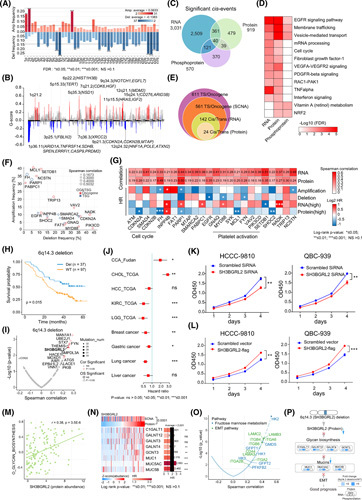
Impacts of SCNAs on mRNA, protein, and phosphoprotein expressions of cholangiocarcinoma (CCA). (A) Arm amplification (red) and deletion (blue) landscape of the FU‐CCA cohort. The significant focal events are labeled in the plot (Fisher's exact test). (B) Significant focal peaks of CCA with annotated driver genes. (C) Overlap of *cis*‐effects observed at mRNA, protein, and phosphoprotein levels (FDR <0.05). (D) Heatmap of significant pathways based on significant *cis*‐events observed at mRNA, protein, and phosphoprotein levels. (E) Overlap of cancer‐associated genes (CAGs) with significant *cis*‐effects observed at RNA, protein, and phosphoprotein levels (FDR <0.05). (F) Comparison of the amplification and deletion frequencies of 24 CAGs with significant *cis*‐effects, with oncogene in red and TSG in blue. The circle size represents the alteration frequency in the cohort. (G) Heatmap revealing the 24 CAGs correlations between SCNAs and their cognate gene products (mRNA expression [up], protein expression [bottom]; top panel). The bottom heatmap represents the impacts of SCNA, mRNA, and protein level alterations on the OS of patients with CCA. Survival Kaplan–Meier analysis is performed and log‐rank tests are employed to identify significant differences. HRs are calculated using the univariate Cox regression model. The asterisks represent the statistical *p* values (*p < 0.05; **p < 0.01; ***p < 0.001). (H) Survival Kaplan–Meier curves of patients with CCA with (blue) or without (orange) 6q14.3 deletion in the FU‐CCA cohort (two‐sided log‐rank test *p* < 0.05). (I) Spearman correlation analysis showing significant *cis*‐effects of coding genes in 6q14.3 deletion. Survival analysis reveals *SH3BGRL2* as the only gene whose cognate mRNA and protein abundance are significantly correlated with the prognosis of patients with CCA with 6q14.3 deletion (log‐rank test). (J) Comparison of the OS based on SH3BGRL2 mRNA expression between our cohort and other cancer cohorts. HRs are calculated using a univariate Cox regression model. HR >1 represents risk factors and HR <1 represents protective factors for survival. Significance is calculated using the log‐rank test. (K) The decreased SH3BGRL2 significantly inhibits cell growth and proliferation in both HCCC‐9810 and QBC‐939 cells. (L) The increased SH3BGRL2 promotes cell growth and proliferation in both HCCC‐9810 and QBC‐939 cells. (M) Spearman correlation analysis between SH3BGRL2 protein abundance and gene set variation analysis (GSVA) score of O‐linked glycan biosynthesis pathway. (N) Heatmap of the associations of significant *O*‐linked glycan biosynthesis‐related proteins with SCNA and protein abundances of SH3BGRL2 (Benjamini–Hochberg adjusted *p* < 0.05). Color indicates the *Z* score of protein abundance in each sample, with the increase in red and the decrease in blue. HRs are represented in the right panel. The *p* values are calculated by two‐sided log‐rank test. (O) Spearman correlation analysis of *O*‐linked glycan biosynthesis pathways and EMT‐related protein abundance. (P) Systematic diagram summarizing the underlying mechanism of patients with CCA harboring 6q14.3 deletion.

Somatic copy number alteration (SCNA) analysis revealed the most frequent gains in chromosomes 1p/q and 8q as well as losses in 4q, 6p/q, 9p/q, 17p, and 21p/q (Figure 2A). Besides previous reported focal alterations at 1p36.11 and 12q21.1,^7^ we also found significant amplification in 1q21.2 and 6p22.2 and deletion in 9p21.3 and 12q24.32 (Figure 2B). We identified 3031, 919, and 570 *cis*‐effects of SCNAs at the mRNA, protein, and phosphoprotein levels, respectively, of which 40 *cis*‐effects were concordant at all three levels (Figure 2C). Pathway analysis revealed the *cis*‐effect genes were mainly enriched in EGFR signaling, membrane trafficking, and vesicle‐mediated transport (Figure 2D; Table S2). SCNAs with the strongest *trans*‐effects were concentrated in 1q, 2p, 3q, and 10q (Figure S6A; Table S2).

We detected 24 out of 611 cancer‐associated genes (CAGs)^18^ with *cis*‐effect at three omic‐levels (Figure 2E), including eight tumor suppressor genes (TSGs) and 16 oncogenes (Figure 2F; Table S2). Survival analysis for the SCNAs of these 24 CAGs and their cognate mRNAs and proteins revealed the association between 24 CAGs and clinical outcomes (Figure 2G; Table S2). We further investigated the *cis*‐effects and prognostic relevance of these 24 CAGs in the Dong dataset^11^ and demonstrated consistent results (Figure S6B).

Survival analysis of arm‐level and focal arm‐level SCNA events revealed that amplifications of 4p, 14p, 16p/q, 1q21.2, and 6p22.2 as well as deletions of 6p21.2 and 6q14.3 were significantly associated with CCA prognosis (Figure S6C). The 6q14.3 deletion was observed in 26.6% of patients with CCA, with better overall survival (OS; Figure 2H). SCNA of *SH3BGRL2* locates on the 6q14.3 had strong *cis*‐effects on both cognate RNAs and proteins, which were associated with favorable OS (Figure 2I,J; Figure S6D,E; Table S2).

We used siRNA to knock down the expressions of SH3BGRL2 in HCCC‐9810 and QBC‐939 cell lines to confirm the association between the decreased SH3BGRL2 and the improved OS for patients with CCA. These two CCA cell lines were transfected with SH3BGRL2 overexpression vectors for comparison. Knocking down the expression of SH3BGRL2 significantly inhibited cell growth, whereas the overexpression of SH3BGRL2 significantly increased cell proliferation rates in both cell lines (Figure 2K,L).

SH3BGRL2 encodes a protein with an Src homology 3 (SH3) domain,^19^ of which the function remains elusive. Spearman correlation analysis revealed that the *O*‐linked glycan biosynthesis had the strongest correlation with SH3BGRL2 protein abundance (Figure 2M; Figure S6F,G; Table S2). In addition, a significant correlation was observed between the *O*‐glycan biosynthesis of mucins and the gene set variation analysis (GSVA) score of EMT (Figure 2N,O), suggesting that the elevation of mucins could enhance the EMT process. Collectively, we found that the 6q14.3 deletion decreased SH3BGRL2 expression, which downregulated the *O*‐linked glycan biosynthesis, ultimately weakening the EMT process, inhibiting the tumor cell growth, and resulting in a good prognosis for patients with CCA (Figure 2P).

### Proteogenomic alterations of CCAs and NATs

**FIGURE 3:**
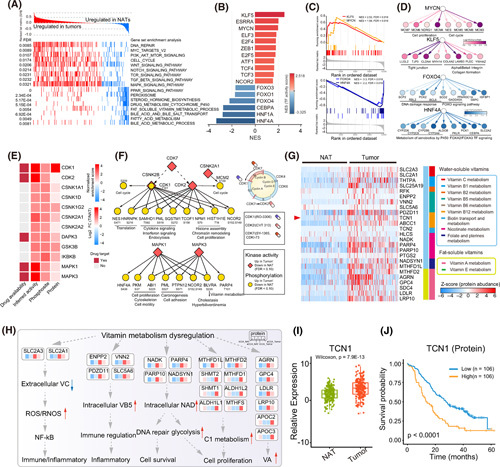
Alterations of transcriptomic, proteomic, and phosphoproteomic profiles of cholangiocarcinoma (CCA). (A) GSEA showing the significantly differentially regulated pathways (FDR <0.05, NES >1.5 and <−1.5) that were significantly enriched in tumors and normal adjacent tissues (NATs). (B) Comparison of TF activity inference from transcriptomic data between tumors and NATs. (C) GSEA plots showing TF activity of KLF5, MYCN, FOXO4, and HNF4A in tumors and NATs. (D) TF‐TG network diagram illustrating differences between tumors and NATs in terms of TGs abundance with their regulating key pathways. (E) Heatmap illustrating kinases of increased activity inferred from phosphorylation of its substrates (normalized enrichment score) or increased phosphorylation of its activating site. Druggable information from DGIdb. (F) Kinase–substrate network diagram illustrating the differences between tumors and NATs in terms of kinase activity and corresponding phosphorylated substrate abundance. (G) Heatmap of significantly differential proteins involved in vitamin metabolisms (Benjamini–Hochberg adjusted *p* < 0.05). The right panel diagram indicates the specific families of vitamin metabolism involving these molecules. (H) Diagram showing the multiomics profiles of vitamin metabolism among tumors and NATs from intrahepatic CCA (iCCA) and extrahepatic CCA (eCCA). (I) Boxplot illustrating TCN1 protein expression in tumors (red) and NATs (green; Wilcoxon rank‐sum test). (J) Survival Kaplan–Meier curves of TCN1 protein expressions, log‐rank test.

Principal component analysis revealed a clear distinction between CCAs and NATs at all three levels (Figure S7A,C,E). Differential gene expression analysis identified 759 mRNAs, 672 proteins, and 484 phosphoproteins that were significantly overrepresented in CCAs (fold change >2; adjusted *p* < 0.05; Table S3). Proteomic‐based gene set enrichment analysis revealed that oncogenic pathways, including cell cycle and DNA repair, were significantly upregulated, whereas PPAR pathway, bile salt transport, and fatty acid metabolism, which are the fundamental functions of BDs, were downregulated in CCAs (Figure 3A; Figure S7B,D,F; Table S3). Particularly, the phosphorylation of RB1_T373, CDK1_T161, and MCM_S27 was identified exclusively in tumors, which were verified by IHC (Figure S2). Noticeably, even though we observed tumor‐concordant upregulated or downregulated pathways at transcriptomic and proteomic levels, the mRNAs and proteins showed distinctive features in regulating CCA tumorigenesis (Figure S7G).

We compared transcription factor (TF) activity inferred from transcriptome (Supporting Materials and Methods) and observed that 11 TFs (KLF5, MYCN, etc.) were significantly activated in CCAs, whereas FOX family and HNF1A/4A activities were downregulated (FDR <0.05; Figure 3B,C; Table S3). By assessing the changes in target genes (TGs), we demonstrated that the TGs of KLF5 and MYCN involved in the tight junction and cell cycle pathways were significantly elevated in CCAs, whereas, the TGs of HNF4A and FOXO4 involved in cytochrome P450 xenobiotic metabolism and DNA damage response pathway were significantly decreased in CCAs (Figure 3C,D). According to phosphoproteomic data, 605 phosphosites had greater changes than cognate protein abundances (Figure S7H). Kinase–substrate enrichment analysis (KSEA) was performed to infer the dominantly activated kinases in CCAs (Supporting Materials and Methods). We detected 13 kinases with significantly increased activity in CCAs, including CDK (CDK1/2/7) and MAPK families (MAPK1/K3) (Figure 3E; Figure S7I; Table S3). Investigation of the CDK and MAPK regulatory networks demonstrated that the phosphorylated substrates, which were associated with the cell cycle, chromatin remodeling, and cell adhesion, were also elevated in CCAs (Figure 3F; Figure S7J,K; Table S3).

Strikingly, we observed that vitamin metabolic molecules were significantly upregulated in CCAs. We further surveyed the vitamin pathway family using the annotation of the Reactome (Figure 3G; Table S3). Key enzymes involved in vitamin C, vitamin B5, nicotinate, folate, vitamin A, and vitamin B12 (VB12) metabolism were significantly upregulated in CCAs (Figure 3H; Table S3). Notably, TCN1 as a critical VB12 transporter was significantly elevated in tumors (Figure 3I) and was associated with poor prognosis at both mRNA and protein levels (Figure 3J; Figure S7L). Accordingly, the prognostic relevance of TCN1 was further confirmed in both TCGA^3^ and Dong^11^ cohorts (Figure S7L–N).

### Proteome intertumoral heterogeneity of CCA and their associations with clinical outcomes

**FIGURE 4:**
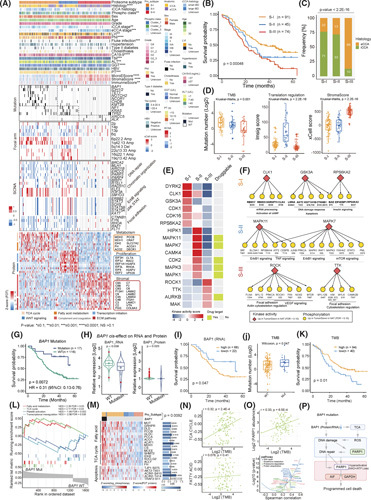
Proteomic stratification of cholangiocarcinoma (CCA) and their associations with clinical outcomes. (A) Unsupervised clustering of tumor samples into three NMF‐derived proteomic subtypes: S‐I (*n* = 93, metabolism subtype); S‐II (*n* = 46, proliferation subtype); and S‐III (*n* = 78, stromal subtype) based on the top 3000 genes with high coefficient variations (CV). The upper heatmap indicates the clinical features (top panel), unsupervised cluster for phosphoproteomic data, and xCell signature scores. The middle heatmap indicates genomic alterations across the three proteomic subtypes, including specific gene mutations, significant arms, focal arm events, and SNAs. The lower heatmap displays protein expression profiles in representative pathways across the three proteomic subtypes. The *p* values for categorical and continuous variables are calculated using Fisher's exact test and Wilcoxon rank‐sum test, respectively. (B) Survival Kaplan–Meier curves of proteomic subtypes (log‐rank test). (C) Stacked bar plot showing the proportions of patient distribution in each proteomic subtype, with intrahepatic cholangiocarcinoma (iCCA) in green and extrahepatic cholangiocarcinoma (eCCA) in orange (Fisher's exact test). (D) Boxplots illustrating TMB, translation regulation score, and stromal score across proteomic subtypes (Wilcoxon rank‐sum test). (E) Heatmap of inferred kinase activity across proteomic subtypes. The graph panel to right annotates the clinical druggable information. (F) Kinase–substrate network diagrams in each proteomic subtype. (G) Survival analysis for patients in *BAP1*‐mutant and *BAP1*‐WT groups in the FU‐CCA cohort (log‐rank test). (H) Boxplots showing *BAP1* expression of mRNA and protein levels in *BAP1*‐mutant and *BAP1*‐WT groups (Wilcoxon rank‐sum test *p* < 0.05). (I) Survival Kaplan–Meier curves of *BAP1* in mRNA expression (log‐rank test). (J) Boxplot illustrating higher TMB in *BAP1*‐mutant than that in *BAP1*‐WT group. (K) Survival Kaplan–Meier curves of patients with CCA with high and low TMB (log‐rank test). (L) GSEA plots of TCA cycle and fatty acid metabolism pathways in *BAP1*‐mutant and *BAP1*‐WT groups. (M) Heatmap showing significant protein expressions in TCA cycle and fatty acid metabolism pathways (Benjamini–Hochberg adjusted *p* < 0.05). HRs are calculated using the univariate Cox regression model. *BAP1* mutation is statistically associated with proteomic subtypes (Fisher's exact test **p < 0.01). (N) Spearman correlation analysis of TMB in TCA cycle and fatty acid metabolism pathways, respectively. (O) Spearman correlation analysis between PARP1 protein expression and TMB (top). Volcano plot shows the correlation of significant pathways with PARP1 protein abundance (bottom). (P) Systematic diagram summarizing the impact of the mechanism underlying patients with *BAP1*‐mutant CCA is associated with a favorable prognosis.

We performed non‐negative matrix factorization‐based unsupervised clustering and classified CCA into three proteomic subtypes (S‐I, S‐II, and S‐III; Figure 4A; Figure S8A; Table S4). Phosphoproteomic clusters also defined three phosphoproteomic subtypes, which significantly overlapped with the proteomic subtypes (*p* < 0.01; Figure 4A; Figure S8E). Among the proteomic subtypes, S‐I (*n* = 93) was characterized by metabolism‐related proteins in the TCA cycle and fatty acid metabolism, defining as the metabolism subtype. S‐II (*n* = 46) was featured by transcription initiation‐related proteins and the WNT signaling pathway, designating as the proliferation subtype. S‐III (*n* = 78) was enriched in ECM and coagulation and complement pathways, denoting the stromal subtype (Figure 4A; Figure S8B).

S‐I had the highest tumor mutational burden (TMB) (Figure 4D; Table S1). *BAP1* and *IDH1/2* were enriched in S‐I, whereas *NF1* and *KRAS* were dominant in S‐II (*p* < 0.05; Figure 4A). Proteomic subtypes were strongly relevant to clinical features, with S‐I having the best and S‐III having the worst OS (Figure 4B; Figure S8C). Notably, eCCAs were significantly enriched in S‐III, whereas iCCAs were mainly enriched in S‐I and S‐II (Fisher's exact test, *p* < 2.2E‐16; Figure 4A,C). By performing clustering analysis utilizing our proteomic subtype–specific signatures, we reproduced the consistent consensus subtypes classifying in the Dong cohort,^11^ which emphasized the robustness of our proteomic subgrouping system (Figure S8D). We identified 17 proteomic subtype–specific kinases by KSEA, including CDK1, CLK1, and GSK3A in S‐I; MPAKs (MAPK1/3/7/11) in S‐II; and ROCK1 and TTK in S‐III (Figure 4F; Table S4), six of which were identified as therapeutic targets (Figure 4E).

### Proteomic characteristics of CCAs with mutations of *BAP1* and *ARID1A*

We identified that *BAP1* mutations were enriched in the S‐I and exclusively in iCCA, with significant relevance to better clinical outcomes (Figure 4G; Figure S9C,D; Table S4). Functionally, BAP1 is a ubiquitin carboxy‐terminal hydrolase involved in regulating DNA damage repair, cell death, and mitochondrial metabolism.^20^

*BAP1* mutation resulted in significantly decreased mRNA and protein (Figure 4H), which were associated with favorable survival (Figure 4I). The same survival trends were further validated in the TCGA‐CHOL (Figure S8H). Compared to the *BAP1*‐WT, patients who were *BAP1*‐mutant had higher TMB (Figure 4J,K) and significantly elevated TCA cycle and fatty acid metabolism (Figure 4L,M; Table S4). Pairwise Spearman correlation analysis indicated that the TCA cycle, but not lipid metabolism, contributed to the increased TMB (Figure 4N). Consistent with the reports that the TCA cycle promotes ROS accumulation in cells, which induces DNA damage,^21^ we found that *BAP1*‐mutant CCAs had significantly higher ROS accumulation than the *BAP1*‐WT (Figure S8F,G).

Importantly, PARP1, which responds to DNA damage (Figure S8K),^22^ was identified to be significantly elevated in the *BAP1*‐mutant group at both mRNA/protein levels (Figure S8I). We found that TMB level and PARP1 protein abundance exhibited a strong positive correlation (Figure 4O). Correspondingly, increased PARP1 was associated with a good prognosis (Figure S8J). Previous studies have shown that persistent PARP1 activation induces cell programmed death.^22^,^23^ We investigated the changes of apoptosis‐associated molecules and observed a higher level of GAPDH as an apoptosis‐inducing factor in the *BAP1*‐mutant than that in the *BAP1*‐WTs (Figure S8L) as well as that TJP1, AKT2, ACIN1, and TRAF2 involved in cell apoptosis were significantly phosphorylated in *BAP1*‐mutant (Figure 4M; Table S4). Our findings suggested that *BAP1* mutation, as a loss of function, upregulated the TCA cycle and initiated DNA damage through ROS accumulation, resulting in PARP1 hyperactivation–induced cell apoptosis (Figure 4P).

Furthermore, *ARID1A* mutations were identified and associated with favorable prognosis (Figure S9A,B). *ARID1A* mutations decreased its cognate protein expression and were associated with the elevation of the mRNAs and proteins that enriched in nucleotide excision repair, purine metabolism, and mitochondrial translation as well as the reduction of mRNAs and proteins that participated in immune response and anti‐inflammatory response (Figure S9E–H).

### The impact of HBV infection on patients with CCA

**FIGURE 5:**
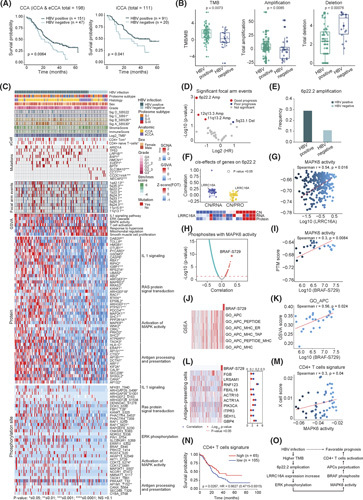
The impact of HBV infection on patients with cholangiocarcinoma (CCA). (A) Overall survival analysis of patients with CCA with and without HBV infection, as well as of patients with intrahepatic cholangiocarcinoma (iCCA) in the FU‐CCA cohort (log‐rank test *p* < 0.05). (B) Comparisons of TMB, total amplification, and total deletion between patients with CCA with and without HBV infection (Wilcoxon rank‐sum test *p* < 0.05). (C) Heatmap visualizing multiomics profiles of patients with CCA with and without HBV infection. (D) Volcano plot showing *p* value versus HR of different focal peaks. The 6p22.2 amplification is labeled in red for its influence on good prognosis. (E) Frequency of 6p22.2 amplification peak in patients with CCA with and without HBV infection. (F) *Cis*‐effects of SCNA on RNA and protein abundance of 6p22.2 amplification peak. *LRRC16A* is the only significant cis‐event on both SCNA–mRNA and SCNA–protein correlation (Spearman correlation *p* < 0.05). *LRRC16A* mutation is positively correlated with its cognate mRNA and protein abundances. (G) Relationship between *LRRC16A* protein expression and MAPK6 kinase activity (Spearman correlation *p* = 0.016). (H) Volcano plot showing the downstream phosphosites for MAPK6 kinase activity. (I) Relationship between BRAF‐S729 and MAPK6 kinase activity (Spearman correlation *r* = 0.3, *p* = 0.0084). (J) Heatmap of BRAF‐S729 with GSEA score of antigen processing and presentation pathways. (K) Relationship between phosphosite BRAF‐S729 (Log10 BRAF‐S729) and antigen processing and presentation (Spearman correlation *r* = 0.56, *p* = 0.024). (L) Heatmap of BRAF‐S729 and proteins from antigen processing and presentation pathways. The Spearman correlation coefficients between phosphosite and proteins are calculated with *p* values and displayed in the log 10 scale. (M) Relationship between MAPK6 kinase activity and CD4^+^ T cell signature (Spearman correlation *r* = 0.3, *p* = 0.04). (N) Survival Kaplan–Meier curves of patients with CCA with high and low levels of CD4^+^ T cells (log‐rank test *p* = 0.0267). (O) Summary of favorable prognosis in patients with CCA‐HBV.

Patients with CCA with HBV infection (CCA‐HBV) have an improved OS (Figure 5A). We incorporated non‐HBV cases from published CCA studies^7^,^9^ to avoid bias caused by the unequal number of patients infected with HBV in our cohort (Supporting Materials and Methods). The combined cohort includes 150 patients who were HBV‐positive and 235 patients who were HBV‐negative. The patients who were HBV‐positive in this combined cohort also had a favorable OS (Figure S10A). Further investigation revealed that patients with CCA‐HBV have a higher incidence of TMB, a higher frequency of SCNA amplifications, and a lower frequency of SCNA deletions (Figure 5B). The GSVA based on proteomic and phosphoproteomic data suggested that the ERK phosphorylation, MAPK activity, and antigen processing and presentation (APCs) were overrepresented in patients with CCA‐HBV (Figure 5C; Table S5).

We found that the most prognostic event with higher frequencies for patients with CCA‐HBV was the 6p22.2 amplification (Figure 5D,E; Table S5), which was confirmed by the combined cohort with the Dong cohort^11^ (Figure S10B,C). After screening the coding genes on this locus, *LRRC16A* showed *cis*‐effects of SCNA on both mRNA and protein abundances (Figure 5F; Table S5).

Previous research suggested a significant relationship between LRRC16A and the ERK/MAPK signaling pathway.^24^ We observed a positive correlation between LRRC16A and the MAPK6 kinase activity (Figure 5G). Importantly, BRAF phosphorylation at S729 was identified as the most prominent phosphosite downstream of MAPK6 activity (Figure 5H,I). Further analysis indicated that BRAF‐S729 abundance was strongly correlated with GSVA scores of the APC (Figure 5J,L). Moreover, consistent with the previous reports that APC could recruit CD4^+^ T cells,^25^ we observed a positive correlation between MAPK6 kinase activity and xCell‐derived CD4^+^ T cells (Figure 5M). Survival analysis revealed that a higher level of CD4^+^ T cells was associated with the favorable OS (Figure 5N). Consistently, these observations were further confirmed in a combined cohort^11^ (Figure S10D,E). Overall, our results suggested the possible mechanism of how HBV infection contributes to the favorable prognosis and implied that patients with CCA‐HBV might benefit more from the immune therapy (Figure 5O).

### Comparative proteogenomic characteristics of iCCA and eCCA

**FIGURE 6:**
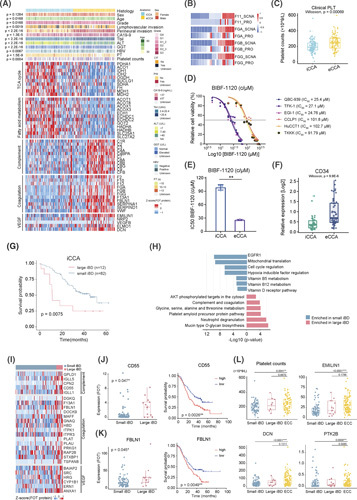
Comparative proteogenomic characteristics of intrahepatic cholangiocarcinoma (iCCA) and extrahepatic cholangiocarcinoma (eCCA). (A) Heatmap visualizing clinical and proteomic profiles of patients with iCCA and eCCA. (B) Protein abundances of F11, FGA, FGB, and FGG are positively correlated with their cognate SCNAs. (C) Boxplots showing a higher level of platelet counts of patients with eCCA than that of patients with iCCA (Wilcoxon sum‐rank test *p* = 0.0004). (D) Dose–response curves of BIBF‐1120 were determined on day 2 after inhibitors adding. The data represent the mean values ± SD (*n* = 3). (E) Half‐maximal inhibitory concentration (IC50) values of BIBF‐1120 were determined on day 2 after inhibitors adding. The data represent the mean values ± SD (*n* = 3). (F) Boxplots showing the comparison of CD34 protein expression between eCCA and iCCA samples. (G) Overall survival analysis of patients with large (red) and small iBD (blue) (log‐rank test *p* < 0.05). (H) KEGG pathway enrichment revealing significantly enriched pathways in patients with large (red) and small iBD (blue). (I) Heatmap showing proteins from complement, coagulation, and VEGF pathways enriched in patients with large iBD. (J) Protein abundance of CD55 from the complement pathway is upregulated in large iBD. Overall survival analysis of patients with cholangiocarcinoma (CCA) with high and low CD55 protein expressions (log‐rank test *p* = 0.0026). (K) Protein abundance of FBLN1 from the coagulation pathway is upregulated in large iBD. Overall survival analysis of patients with CCA with high and low FBLN1 protein expressions (log‐rank test *p* = 0.0049). (L) Boxplots showing platelet counts among small iBD, large iBD, and eCCA, as well as protein abundance of EMILIN1, DCN, and PTK2B from the VEGF signaling pathway (Mann–Whitney *U* test).

To nominate eCCA‐specific druggable targets, we compared molecular features and clinical characteristics of iCCAs and eCCAs (Figure 6A; Figure S11C–F; Table S6). We observed high mutational frequencies of *BAP1*, *KMT2B*, and *KMT2D* in iCCA as well as *THAP9*, *SEC24B*, and *CAND1* in eCCA. The focal SCNAs at 1q21.2, 1q42.13, and 8q24.3 were centered in iCCA, whereas those at 8p11.22, 6q14.3, and 2q11.2 were centered in eCCA (Figure S11A,B). Pathway enrichment analysis indicated that TCA cycle and fatty acid metabolism were enriched in iCCA, whereas complement and coagulation were enriched in eCCA (Figure 6A; Table S6).

We observed 37 significant cis SCNA–protein cascades centered in eCCA (Spearmen correlation, *p* < 0.05), of which the abundances of four proteins (F11, FGA/B/G, complement and coagulation) were positively correlated with their cognate SCNAs (Figure 6B; Figure S11G; Table S6), which is consistent with the high level of platelet counts in eCCAs (Figure 6C).

We performed correlation analysis and found that GSVA scores, as well as the key proteins of the VEGF pathway, were highly associated with FGA/B/G protein expressions (Figure S11H,I; Table S6), which implied the activation of coagulation cascade and angiogenesis. These results illustrated that the eCCAs might be more sensitive to VEGF inhibitors. In support of this hypothesis, we evaluated the drug responses of human eCCA (EGI‐1) and iCCA (ETK‐1, HuCCT1) cells lines to eight VEGF/VEGFR inhibitors (Brivanib, Linifanib, BIBF‐1120, Ponatinib, Cediranib, Sorafenib, Foretinib, and Tivozanib) from the drug database,^26^ and area under the curve of eCCA was significantly higher than those of iCCA (Mann–Whitney U test, *p* = 0.0186 and 0.0194; Figure S11J). We collected six CCA cell lines, including three iCCA (HuCCT1, CCLP1, and TKKK) and three eCCA (QBC‐939, TFK‐1, and EGI‐1), and treated them with three VEGF/VEGFR inhibitors (Brivanib, BIBF‐1120, and Cediranib), *in vivo*. Consequently, eCCA cell lines were more sensitive to the VEGF/VEGFR inhibitors with lower IC50 values (Figure 6D,E; Figure S11K,L). We estimated the vascular density between eCCAs and iCCAs using the vascular endothelial marker CD34 because the VEGF signaling pathway has been reported to promote the proliferation of CD34^+^ vascular endothelial cells. As expected, the protein expression of CD34 was significantly higher in eCCAs than iCCAs (Figure 6F), which was further confirmed by IHC (Figure S11M).

### Distinctive molecular features between large versus small duct subtypes

The genomic and proteomic diversities of large iBD and small iBD were still unknown. The survival of patients with large iBD was worse than patients with small iBD (Figure 6G), which is consistent with previous reports.^27^

At the genomic level, the mutational frequencies of 53 genes showed significant differences between large iBD and small iBD, of which *SMAD4* and *SETBP1* were covered by IntOGen (Figure S12A,B).^18^ *SMAD4* resulted in concordantly increased mRNAs and proteins (Figure S12C,D) and was associated with the alteration of the mucin synthesis–related pathways and the immune pathways (Figure S12E,H). Conversely, 6q and 6q14.3 were deleted predominantly in small iBD, of which 6q14.3 have a relatively better prognosis (Figure S12I–K).

At the proteomic level, the small iBD was characterized by EGFR and mitochondrial translation, whereas large iBD was dominant in mucin‐type *O*‐glycan biosynthesis, complement and coagulation, which demonstrated that large iBD was more similar to eCCA than small iBD (Figure 6A,H; Table S6). Proteins enriched in complement and coagulation, as well as VEGF pathways (CD55, FBLN1, BAIAP2), were upregulated in large iBD (Figure 6I; Table S6). The patients with higher expression of CD55 and FBLN1 had worse survival (Figure 6J,K). Consistent with eCCAs, the platelet counts were significantly higher in large iBD than in small iBD. This phenomenon was also represented by the critique proteins (EMILIN1, DCN, and PTK2B) on VEGF pathway (Figure 6L).

### Characterization of immune infiltration in CCA

**FIGURE 7:**
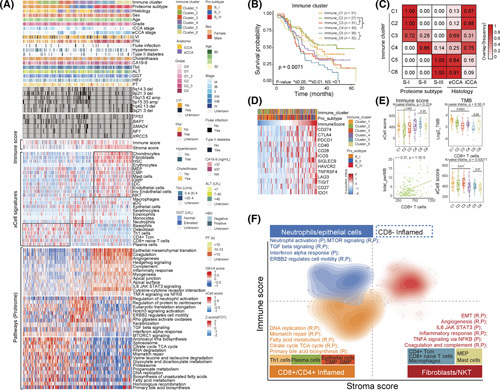
Immune landscape cholangiocarcinoma (CCA). (A) Heatmap illustrating the immune infiltration features and activities of significant pathways across six immune clusters. The bottom panel shows the selected significant clinical features, proteomic subtypes, and genomic alterations of mutations and focal arm events. The second graph panel shows deconvoluted immune/stromal signatures using xCell algorithm based on proteomic data (STAR Methods), together with the derived relative abundance of immune and stromal cell types. The bottom heatmap panel shows some key upregulated pathways across different immune clusters using the gene set variation analysis (GSVA) R package based on multiomics profiles (“common”) or only global protein abundance (Wilcoxon rank‐sum test FDR <0.01). (B) Survival Kaplan–Meier curves of 6 immune clusters (log‐rank test). (C) Heatmap illustrating the overlap of immune clusters with proteomic subtypes and anatomical locations. (D) Significant correlations between ESTIMATE immune scores and immune checkpoint molecules (Spearman correlation). (E) Boxplots illustrating the ESTIMATE immune score, TMB, and CD8^+^ T cell infiltration across different immune clusters (Kruskal‐Wallis test). Spearman correlation analysis of TMB level and CD8^+^ T cell infiltration (bottom left panel). (F) Two‐dimensional density contour plots of different immune clusters based on stromal (*x* axis) and immune (*y* axis) scores deconvoluted from ESTIMATE. For each immune cluster, the significantly upregulated pathways at 10% false discovery rate (FDR) are annotated in the figure based on RNA‐seq (R) and global proteomic (P).

We performed a cell type deconvolution analysis based on proteomic data using xCell to infer the CCA tumor microenvironment (TME; Figure S13C; Table S7; Supporting Materials and Methods). Consensus clustering based on inferred cell type proportions defined six immune subtypes (C1–C6; Figure 7A; Figure S13A; Table S7), which were significantly correlated with OS (Figure 7B). Interestingly, they were strongly overlapped with proteomic subtypes (Figure 7A,C). C1–C3 significantly overlapped with S‐I, enriching CD4^+^ Tcm and CD8^+^ naïve T (adjusted *p* < 0.05). C4 showed a high overlap with S‐II, exhibiting higher infiltrations of epithelial cells and neutrophils. C5–C6 predominantly overlapped with S‐III, which was characterized by enrichments in fibroblasts, endothelial, and NKT cells (Figure 7A,C). Figure 7F summarizes the salient features of each CCA immune cluster.

The ESTIMATE illustrated that the high immune score tended to predict a consistently high expression of immune checkpoint molecules at the mRNA level (Figure 7D; Figure S13B; Table S7). We observed that the immune score and TMB in C1 were significantly higher than those in C2 and C3. Although C4 exhibited a high immune score, the TMB and CD8^+^ T cell infiltration were relatively low. Compared to C6, C5 exhibited a higher infiltration of CD4^+^ and CD8^+^ naïve T cells and a higher immune score (Figure 7E; Table S7). In conclusion, TME analysis revealed a refined clinical stratification of proteomic subtypes into six distinct immune subtypes with different cell type enrichment and nominated that C1 and C5 might respond to ICI therapy.

### TCN1 promotes tumor cell growth through enhancing VB12 metabolism

**FIGURE 8:**
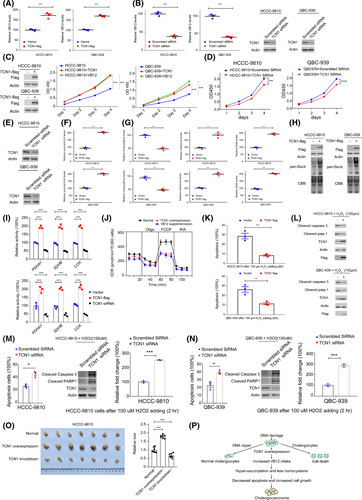
TCN1 promotes tumor cell growth through enhancing VB12 metabolism. (A) TCN1 overexpression notably increases intracellular VB12 levels in human intrahepatic cholangiocarcinoma (iCCA; HCCC‐9810) and extrahepatic cholangiocarcinoma (eCCA) cell lines (QBC‐939). (B) TCN1 knockdown sharply decreases intracellular VB12 levels in HCCC‐9810 and QBC‐939. (C) Enhanced cell growth by TCN1 overexpression and VB12 supplementation in HCCC‐9810 and QBC‐939. (D) The decreased TCN1 significantly inhibited cell growth and proliferation in both HCCC‐9810 and QBC‐939 cells. (E) Western plots showing the decreased TCN1 expressions in both HCCC‐9810 and QBC‐939 cells, after siRNA knockdown. (F) TCN1 overexpression increases succinyl‐CoA levels in HCCC‐9810 and QBC‐939. (G) TCN1 overexpression reduces homocysteine and increases methionine and S‐adenosylmethionine (SAM) levels in HCCC‐9810 and QBC‐939. (H) TCN1 overexpression induces hypersuccinylation. CBB, Coomassie brilliant blue. (I) Enzymatic activities of PDHA1, SDH, and COX, are inhibited by TCN1 overexpression but are activated by TCN1 knockdown in HCCC‐9810 and QBC‐939. (J) TCN1 overexpression inhibits the OCR of HCCC‐9810. (K) TCN1 overexpression significantly reduces H_2_O_2_‐induced apoptosis. (L) TCN1 overexpression decreases H_2_O_2_‐induced cleaved caspase 3 and cleaved PARP. (M) Decreased TCN1 significantly increased the apoptosis induced by H_2_O_2_ in both HCCC‐9810 cells, and the increased cleaved caspase 3 and cleaved PARP induced by H_2_O_2_ in TCN1 knockdown HCCC‐9810 cells. (N) Decreased TCN1 significantly increased the apoptosis induced by H_2_O_2_ in both QBC‐939 cells, and the increased cleaved caspase 3 and cleaved PARP induced by H_2_O_2_ in TCN1 knockdown QBC‐939 cells. (O) TCN1 overexpression promotes the xenograft growth of tumor cells, whereas TCN1 knockdown delays the xenograft growth of tumor cells. (P) Working model. Increased TCN1 enhanced the transport of VB12 and promoted tumor growth through inhibiting cell apoptosis.

Proteomic analysis revealed dysregulated expression levels of enzymes in VB12 metabolism, in which TCN1 was the most significant one in tumors (Figure 3I), and predicted poor survival (Figure 3J). TCN1 facilitates VB12 transport into cells. From the GEPIA, we found the low TCN1 expression level in normal BD (Figure S14A), suggesting that TCN1 overexpression enhanced VB12 absorption in CCAs, thus promoting tumor growth. We validated that TCN1 overexpression notably increased intracellular VB12 levels in human iCCA and eCCA cell lines (HCCC‐9810 and QBC‐939; Figure 8A), whereas TCN1 knockdown sharply decreased intracellular VB12 levels (Figure 8B). By utilizing 5‐ethynyl‐2′‐dexyurdine (EDU) staining to monitor DNA synthesis, we found that TCN1 overexpression (Figure S14B) and VB12 supplementation (Figure S14C) increased DNA synthesis. In contrast, TCN1 knockdown decreased DNA synthesis (Figure S14D). The proliferation of CCA cell lines was enhanced by TCN1 overexpression and VB12 supplementation and decreased by knocking down TCN1 (Figure 8C–E). In cells with high levels of endogenous TCN1 expression, including gastric epithelial and human nonsmall cell lung cancer cell lines (GES1 and HCC827), we found that TCN1 overexpression or VB12 supplementation did not promote cell proliferation (Figure S14D). These results indicated that the growth and proliferation of CCA cells were easily enhanced by increasing TCN1 because of the low expression of TCN1 in normal BD.

We next surveyed how TCN1 overexpression and VB12 supplementation promote cell proliferation. VB12 plays a key role in methionine and succinyl‐CoA metabolisms (Figure S14E). In the methionine metabolism, VB12 promoted homocysteine removal and methionine production (Figure S14F). We validated that TCN1 overexpression significantly reduced homocysteine and increased methionine and S‐adenosylmethionine (Figure 8F). In the succinyl‐CoA metabolism, we found that TCN1 overexpression increased succinyl‐CoA levels (Figure 8G). Accordingly, we observed that TCN1 overexpression induced hypersuccinylation, as detected using the anti‐pan‐succinyllysine antibody (anti‐SucK) (Figure 8H). In contrast, TCN1 knockdown decreased succinyl‐CoA and pan‐lysine‐succinylation levels (Figure S14G). We previously found that hypersuccinylation regulated metabolic enzyme activities. Here, we validated that TCN1 overexpression inhibited, whereas TCN1 knockdown activated, the enzymatic activities of the catalytic subunits of pyruvate dehydrogenase complex (PDHA1), succinate dehydrogenase (SDH), and cytochrome c oxidase (COX) (Figure 8I). Meanwhile, TCN1 overexpression inhibited the oxygen consumption rate (OCR) (Figure 8J). High homocysteine level induces cell apoptosis, and hypersuccinylation is the major contributor to apoptosis resistance in tumor cells. In accordance, we found TCN1 overexpression reduced (Figure 8K,L), whereas TCN1 knockdown increased (Figure 8M,N), the cell apoptosis in H_2_O_2_ treated cell lines. Increased TCN1 expression promoted the xenograft growth of tumor cells, and TCN1 inhibition delayed the xenograft growth of tumor cells (Figure 8O). Taken together, we confirmed that increased TCN1 enhanced VB12 transport and promoted tumor growth through the inhibition of cell apoptosis (Figure 8P).

## DISCUSSION

Although HBV infection has been proposed as a CCA risk factor, its impacts on prognosis have been controversial.^2^ We observed that patients with CCA‐HBV were associated with prolonged survival. Integrative analysis revealed that patients infected with HBV showed elevated CD4^+^ T cell infiltration, higher amplification frequencies, and lower deletion frequencies, which might be caused by the fact that the HBV virus could insert into the host genome^28^ and replicate. The amplification of *LRRC16A* (located on 6p22.2) could activate the MAPK6 signaling pathway and enhance APC processes through phosphorylating signaling transduction in patients infected with HBV. These results uncovered that HBV infections were associated with increased CD4^+^ T cell infiltration and implied the probability of immune therapy for patients with CCA‐HBV.

Multiomics analysis revealed the profound differences between iCCAs and eCCAs, especially for the elevated VEGF signaling pathway in eCCAs. This phenomenon might be associated with the amplification frequencies of complement and coagulation components (FGA/B/G), which were further proved to activate the VEGF signaling pathway in eCCAs through *cis*‐effect. Our findings suggested that patients with eCCA might be more sensitive to RTK therapy. As the large iBD were similar to eCCA, the VEGF/VEGFR inhibitor might also be more effective for large iBD than for small iBD.

We performed multiomics subtyping based on the proteome, phosphoproteome, and deconvoluted immune scores. Proteomic subtyping significantly overlapped with phosphoproteomic subtyping, implying the concordance between protein expression panel and signaling transduction. We also nominated the potential druggable targets for each subtype based on the featured kinase of each subtype. Interestingly, combined with immune features‐based clustering, we found the three proteomic subtypes could be further classified into six immune subtypes, indicating that patients with similar proteomic or phosphorylating patterns could present diverse immune microenvironments. Proteomic subtype S‐I could be further classified into immune subtypes C1 and C3, in which C1 showed elevated immune scores and might be more beneficial from ICI treatment. The cause of diverse microenvironments in a certain proteomic subtype deserves further investigation.

Other vitamin molecules, including vitamin K2 and vitamin D3, have been proven to associate with CCA tumor progression, whereas the interaction between VB12 and CCA is poorly reported. Functionally, VB12 is bound to haptocorrin and transcobalamin in blood, the former being encoded by TCN1 and the latter by TCN2.^29^ A recent study has revealed that TCN1 is highly expressed in most colon cancer tissues both at transcription and translation levels, whose upregulation is associated with tumorigenesis and progress. Therefore, inhibiting the expression of TCN1 might increase cancer cell apoptosis and restrain its proliferation, thus making it a promising therapy in CCA.

## Supplementary Material
