# Supplementary material for: Proteogenomic characterization of cholangiocarcinoma
Source: Hepatology. 2022 Jul 5;77(2):411–29. doi: 10.1002/hep.32624 (PMC9869950; doi:10.1002/hep.32624)
Supplement: Supplementary file 1 [file hep-77-411-s001.docx]

**Supporting Materials and Methods**

**Proteogenomic Characterization of Cholangiocarcinoma**

Mengjie Deng^1, #^, Peng Ran^1, #^, Lingli Chen^2, #^, Yunzhi Wang^1, #^, Zixiang Yu^2, #^, Ke Cai^1, #^, Jinwen Feng^1^, Zhaoyu Qin^1^, Yanan Yin^1^, Subei Tan^1^, Yang Liu^1^, Chen Xu^2, *^, Guoming Shi^3, *^, Yuan Ji^2, *^, Jian-Yuan Zhao^5, *^, Jian Zhou^3,4 *^, Jia Fan^3,4 *^, Yingyong Hou^2, *^, Chen Ding^1, *^

^1^State Key Laboratory of Genetic Engineering and Collaborative Innovation Center for Genetics and Development, School of Life Sciences, Institute of Biomedical Sciences, Human Phenome Institute, Zhongshan Hospital, Fudan University, Shanghai 200433, China;

^2^Department of Pathology, Zhongshan Hospital, Fudan University, Shanghai 200032, China.

^3^Department of Liver Surgery and Transplantation, Liver Cancer Institute, Zhongshan Hospital, Fudan University, and Key Laboratory of Carcinogenesis and Cancer Invasion of Ministry of Education, 180 Fenglin Road, Shanghai 200032, China

^4^Key Laboratory of Medical Epigenetics and Metabolism, Institutes of Biomedical Sciences, Fudan University, Shanghai 200032, China

^5^Institute for Development and Regenerative Cardiovascular Medicine, MOE-Shanghai Key Laboratory of Children’s Environmental Health, Xinhua Hospital, Shanghai Jiao Tong University School of Medicine, Shanghai 200092, China.

^*^To whom correspondence should be addressed. Email: xu.chen@zs-hospital.sh.cn (C.X), shi.guoming@zs-hospital.sh.cn (G.M.S), ji.yuan@zs-hospital.sh.cn (Y.J), zhaojy@fudan.edu.cn (J.Y.Z.), zhou.jian@zs-hospital.sh.cn (J.Z), fan.jia@zs-hospital.sh.cn (J.F), hou.yingyong@zs-hospital.sh.cn (Y.H.), chend@fudan.edu.cn (C.D.)

^#^ These authors contribute equally

**Supporting Materials and Methods**

**Clinical sample preparation**

**Patient cohorts and clinical data**

We performed a systematic review of the case records and diagnostic materials of CCA patients who underwent primary curative resection from January 2012 to December 2018 at Zhongshan Hospital of Fudan University. Among these patients, 326 patients were diagnosed with CCA. We retrieved and scanned all the available diagnostic pathological slides. All the histological and H&E slides were independently reviewed by at least two experienced pathologists (L.L.C., Z.X.Y., and Y.Y.H.) to verify the diagnostic results. CCA patients on prior anticancer treatments were excluded from this study. Then, 217 cases with CCAs and paired NATs were randomly recruited as the CCA cohort for further research analysis, including 114 iCCA and 103 eCCA patients. The clinical information regarding patient history, the status of surgery along with relevant diagnostic information and survival records were obtained from Zhongshan Hospital and the baseline characteristics of CCA patients were summarized in **Table S1**. The Research Ethics Committees of Zhongshan Hospital, Fudan University approved this study (B2019-200R) and written informed consent were obtained from all the involved patients.

**Sample preparation**

FFPE specimens were prepared and provided by Zhongshan Hospital. A 4 μm slide from each FFPE block was used for H&E staining. For genomic, proteomic, and phosphoproteomic sample preparation, 10 μm slides were deparaffinized with xylene and washed with gradient ethanol. For RNA sample preparation, 10μm slides were prepared from the samples without xylene deparaffination or gradient ethanol wash. The specimens were selected according to H&E staining status and scraped. All materials were aliquoted and stored at −80℃. Each sample was assigned a new research ID and the patient pathology reports were de-identified.

**Combined cohorts**

Three independent cohorts with HBV infection status and molecular characteristics were recruited for the analysis of HBV-CCA in our study. Clinical features of non-HBV patients from the ICGC and Zou et al. cohorts^(1, 2)^ were incorporated to perform the survival analysis. The genomic and transcriptomic data on non-HBV patients from another study^(3)^ were included to confirm our findings regarding molecular characteristics of HBV-positive patients that might impact patients' overall survival.

**Tumor cellularity**

Histology of the tumors and NATs was examined using H&E staining and evaluated independently by at least two board-certified experienced pathologists. Information regarding tumor histological subtype was determined by the 8th edition of the AJCC cancer staging system. Tumor samples were then macro-dissected according to pathological defined tumor enriched area to further elevate the tumor cellularity. As a result, the tumor samples used for follow-up multi-omics analysis were characterized by histologic tumor cellularity ranging from 67% to 89% (median 79%). Tumor cellularity was further independently evaluated in SCNA data on the 139 tumor tissues, using the ABSOLUTE algorithm. Accordingly, the tumor cellularity validated by ABSOLUTE ranged from 70% to 90% (median 81%), which is concordant with histologically assessed tumor cellularity (**Table S1**).

**Cell lines and treatments**

Human CCA cell lines (iCCA: HCCC-9810, CCLP1, HuCCT1, and TKKK; eCCA: QBC-939, TFK-1, EGI-1; Cells were purchased from DSMZ and ATCC) were incubated in Dulbecco’s modified Eagle’s medium (DMEM; high glucose; Thermo. USA) medium with 10% fetal bovine serum (FBS), 100 U/ml penicillin, and 100 U/ml streptomycin in an incubator with 5% carbon dioxide (CO_2_) at 37 °C.

**Whole-exome sequencing**

***DNA extraction and library preparation***

DNA isolated from FFPE tumor samples was used for WES and matched germline DNA was obtained from FFPE NAT samples. DNA was isolated from FFPE samples using DNeasy Blood & Tissue Kit (Qiagen, 69504) according to the manufacturer’s instructions. Purified DNA was quantified using a Qubit 3.0 Fluorometer (Life Technologies). For matched germline and tumor tissues, 100 ng of DNA was sheared to 200–300-bp fragments using a Covaris M220 system. Tumor and matched germline DNA libraries were constructed using Accel-NGS 2 S HYB DNA LIBRARY KIT (Swift Biosciences, 23096) and Accel-NGS 2 S MID S1-S4 (Swift Biosciences, 279384). xGen Exome Research Panel v1.0 (IDT, 1056115) and xGen Lockdown reagents (IDT, 1072281) were used for exome enrichment. Dynabeads M-270 Streptavidin (Thermo, 65306) was used for library purification, P5/P7 primers (Nanodigmbio, ND10010), and HotStart ReadyMix (KAPA, KK2612) were used for library amplification. The amplified libraries were purified using SPRISELECT (Beckman, B23319). The DNA quality was assessed using a Bioanalyzer High Sensitivity DNA Analysis kit (Agilent Technologies, 5067-4626). Samples underwent paired-end sequencing on the Illumina NovaSeq 6000 platform, with a 150-bp read length. The WES target region was 33 M.

***Clustering and sequencing***

The index-coded sample clustering was performed on a cBot Cluster Generation System using Hiseq PE Cluster Kit (Illumina) according to the manufacturer’s instructions.

***Whole-exome sequencing quality control***

The original fluorescence image files obtained from the NovaSeq platform were transformed into short reads (raw data) by base calling and recorded in FASTQ format, which contained sequence information and corresponding sequencing quality information. Sequence artifacts, including reads containing adapter contamination, low-quality nucleotides, and unrecognizable nucleotide^(4)^, undoubtedly set the barrier for the subsequent reliable bioinformatic analysis. Hence quality control was essential to guarantee meaningful downstream analysis.

The steps of data processing were as follows:

1. Discard the paired reads if one read contained adapter contamination (> 10 nucleotides aligned to the adapter, allowing ≤ 10% minima match).
2. Discard the paired reads if > 10 % of bases were uncertain in either one read.
3. Discard the paired reads if the proportion of low-quality (Phred quality < 5) bases was > 50 % in either one read.

All the downstream bioinformatic analyses were based on high-quality clean data, which were retained after these steps. Simultaneously, quality control (QC) statistics including total reads number, raw data, raw depth, sequencing error rate, percentage of reads with Q30 (the percent of bases with Phred-scaled quality score > 30), and GC content distribution were calculated and summarized.

**RNA sequencing**

***RNA extraction and RNA quantification***

RNA was extracted from tissues by using TIANGEN® RNAprep Pure FFPE Kit (Catalog: DP439) according to the reagent protocols. All RNA analytes were assayed for RNA integrity, concentration, and fragment size. Samples for total RNA-seq were quantified on a TapeStation system (Agilent Technologies).

***Total RNA-seq library construction***

For RNA sequencing library preparation, 500 ng RNA per sample was used as the input material. Sequencing libraries were generated using Ribo-off® rRNA Depletion Kit (H/M/R) (Vazyme, Catalog: N406) and VAHTS® Universal V6 RNA-seq Library Prep Kit for Illumina (Catalog: N401-NR604) following the manufacturer’s directions and index codes were added to attribute sequences to each sample.

***Total RNA sequencing***

The indexed libraries were sequenced on an Illumina platform and 150 bp paired-end reads were generated. Typically, these were pooled of four samples. The raw Illumina sequence data were demultiplexed and converted to FASTQ files, and the adapter and low-quality sequences were quantified. The samples were then assessed for QC by mapping reads to the hg38 human genome reference, estimating the total number of mapped reads, amount of mapped RNA to coding regions, amount of rRNA in the sample, number of genes expressed, and relative expression of housekeeping genes. The samples passing this QA/QC were then clustered with other expression data from similar and distinct tumor types to confirm the expected expression patterns. Atypical samples were then SNP typed from the RNA data to confirm the source analyte. FASTQ files of all reads were then uploaded to the NODE repository (<http://www.biosino.org/node>).

**Peptide preparation for MS analysis**

***Protein extraction and digestion***

To prepare peptides for MS analysis, 10 μm slides from FFPE blocks were macro-dissected, deparaffinized with xylene, and washed with ethanol. Then, 100 μL TCEP buffer (2% deoxycholic acid sodium salt, 40 mM 2-chloroacetamide, 100 mM tris-phosphine hydrochloride, 10 mM [2-carboxyl)-phosphine hydrochloride, and 1 mM phenylmethylsulfonyl fluoride mixed with MS water, pH 8.8) was added into a 1.5 mL tube with prepared samples and heated in a metal bath at 99℃ for 30 min. It was then cooled to ambient temperature, 3 μg trypsin was added into each tube, and digested in a 37℃ incubator for 14 h.

***Peptide desalination***

Then, 26 μL 10% formic acid (FA) was added to each tube, vortexed for 3 min, and then centrifuged at 12,000× g for 10 min. The supernatant was collected in a new 1.5 mL tube with 350 μL buffer (0.1% FA in 50% acetonitrile [ACN]) and extracted by a vortex for 3 min and centrifuging at 12,000× g for 5 min. The supernatant was transferred into a new tube for drying in a vacuum drier at 60℃. Then, 100 μL 0.1% FA was added for dissolving the peptides, vortexed for 3 min, and centrifuged at 12,000× g for 5 min. The supernatant was collected in a new tube and then desalinated. Before desalination, the pillars were activated with 2 slides of 3M C8, and the lipids involved were: 100 μL 100% ACN twice, 100 μL 50% ACN once, and 100 μL 0% ACN twice. After pillar balance with 100 μL 0.1% FA twice, the supernatant was loaded in the pillar twice, and decontaminated with 100 μL 0.1% FA twice. Lastly, 100 μL elution buffer (0.1% FA in 50% ACN) was added into the pillar for elution twice and only the effluent was collected for MS. The collected liquid was evaporated to dryness in a vacuum drier at 60℃ and stored at −80℃ until LC-MS/ MS analysis.

***Phosphopeptide enrichment***

For the phosphoproteomic analysis, the peptides were extracted from the FFPE slides after trypsin digestion using the methods described above. The tryptic peptides were then enriched with High-Select™ Fe-NTA Phosphopeptides Enrichment Kit (Thermo Fisher Scientific) following the manufacturer’s recommendations. Briefly, the peptides were suspended with binding/wash buffer (provided in the enrichment kit), mixed with the equilibrated resins, and incubated at 21−25℃ for 30 min. After incubation, the resins were washed thrice with binding/wash buffer and twice with water. The enriched peptides were eluted with elution buffer (contained in the enrichment kit) and dried in a vacuum drier at 30℃.

**ESI-LC-MS/MS analysis**

***Proteome and phosphoproteome analysis with Liquid chromatography–tandem mass spectrometry***

The Orbitrap Exploris 480 Mass Spectrometer (Thermo Fisher Scientific) is equipped with an Easy nLC-1200 (Thermo Fisher Scientific) and a Nanoflex source (Thermo Fisher Scientific). The peptides were re-dissolved in 12 μL loading buffer (5% methanol and 0.2% FA). Peptide samples were loaded onto a trap column (100 μm × 2 cm, homemade; particle size, 3 μm; pore size, 120 Å; SunChrom, USA), separated by a homemade silica microcolumn (150 μm× 30 cm, particle size, 1.9 μm; pore size, 120 Å; SunChrom, USA) with a gradient of 4–100% mobile phase B (80% acetonitrile and 0.1% formic acid) at a flow rate of 600 nL min^−1^ for 150 min.

LC–MS/MS based proteomic and phosphoproteomic experiments were conducted with Field Asymmetric Ion Mobility Spectrometry (FAIMS). FAIMS voltages were set to −45 V and −65 V, respectively, and other parameters were consistent and set as follows: protein quantification consisted of an MS1 scan at a resolution of 120,000 (at 400 m/z). The automatic gain control (AGC) for full MS and MS/MS was set to 3E6 and 5E4, respectively, with maximum ion injection times of 80 and 22 ms, respectively.

**Database searching for proteomic and phosphoproteomic MS raw data**

***Peptide identification and protein quantification***

Peptide identification was processed with the one-stop proteomic cloud platform “Firmiana”^(5)^ against the homo sapiens RefSeq protein database (updated on 04-07-2013) in the National Center for Biotechnology Information. The maximum number of missed cleavages was set to two. The mass tolerance allowed for precursor and production was 20 ppm and 0.05 Da, respectively. The fixed modification was carbamidomethyl (C), and the variable modifications were N-acetylation and methionine oxidation. For quality control of protein identification, a target-decoy-based strategy was applied to control the FDR of both peptides and proteins to < 1%. Percolator was used to obtain the probability value (q-value) and validate that the FDR (measured by the decoy hits) of every peptide-spectrum match (PSM) was < 1%. Thereafter, all peptides shorter than seven amino acids were removed. The cutoff ion score for peptide identification was 20. The PSMs in all fractions were combined for protein quality control, which was more stringent. The q-values of both target and decoy peptide sequences were dynamically increased until the corresponding protein FDR was < 1% using the parsimony principle. Finally, to reduce the false-positive rate, proteins with at least one unique peptide were selected for further investigation.

For phosphoproteomic data, a label-free based quantification analysis was performed using Proteome Discover (version 2.3). The maximum number of missed cleavages was set to 2. The mass tolerance allowed for precursor and production was 20 ppm and 0.05 Da, respectively. The fixed modification was carbamidomethyl (C), and the variable modifications were oxidation (M), acetylation (protein N-term), and phospho (S/T/Y). The cutoff FDR, using a target-decoy strategy, was set at 1% for both the proteins and peptides.

***MS quantification of proteins and phosphoproteins***

For the proteomic data, Firmiana was employed for protein quantification, and both the results and raw data from the mzXML file were loaded. Next, for each identified peptide, the extracted-ion chromatogram (XIC) was extracted by searching against the MS1 based on its identification information, and the abundance was estimated by calculating the area under the extracted XIC curve. For calculating protein abundance, the non-redundant peptide list was used to assemble the proteins by following the parsimony principle. Thereafter, the protein abundance was estimated using a traditional label-free, intensity-based absolute quantification (iBAQ) algorithm, which divided the protein abundance (derived from identified peptide intensities) by the number of theoretically observable peptides.^(6, 7)^

For the phosphoproteomic data, the phosphopeptide intensities were extracted using Proteome Discover (version 2.3). For calculating phosphoprotein abundance, the non-redundant phosphopeptide list was used to assemble the proteins by following the parsimony principle. Next, the phosphoprotein abundance was estimated using a traditional label-free, iBAQ algorithm, which divided the protein abundance (derived from the identified peptide intensities) by the number of theoretically observable peptides.^(6)^ For phosphosite localization, the ptmRS^(8)^ was used to determine phosphosite confidence. Phosphosite probability > 0.75 was considered as confident phosphosites.

**Quality control of the MS data**

***Quality control of the MS platform***

For the quality control of MS performance, the HEK293T cell (National Infrastructure Cell Line Resource) lysate was measured every three days as the quality control standard. The standard was digested and analyzed using the same method and conditions as the CCA samples. A pairwise Spearman correlation coefficient was calculated for all quality control runs in the statistical analysis environment R (version 4.1.0), and the results were shown in **Fig. S1D** and **S1E**. The average correlation coefficients of proteome and phosphorylated proteome standards were 0.94 (95% CI: 0.93–0.96) and 0.93 (95% CI: 0.90–0.97), respectively, exhibiting good reproducibility for repeat experiments with the same samples. The above results demonstrated the consistent stability of the MS platform.

***Quality evaluation of proteomic and phosphoproteomic data***

Density plots of peptide counts and protein identifications showed a clear bimodal distribution, indicating that some samples had insufficient peptide or protein yields and should be excluded from further analysis. Therefore, the quality of the generated data was assessed by examining the distribution status of the identified proteins and phosphorylated peptides for all samples in the R environment (version 4.1.0). In our cohort, 217 CCAs and 197 NATs passed quality control and were used for further proteomic analysis. All samples passed the quality control and showed excellent consistency in terms of proteome quantification (**Fig. S1C**), exhibiting a typical unimodal (Gaussian or normal) distribution (dip statistical test).

**Whole-exome sequencing data analysis**

***Reads mapping & genomic variant calling***

Valid sequencing data were mapped to the reference human genome (UCSC hg19) using the Burrows-Wheeler Aligner (BWA) software to get the original mapping results stored in BAM format.^(9, 10)^ If one or one paired read(s) were mapped to multiple positions, the strategy adopted by BWA was to choose the most likely placement. If two or more most likely placements were presented, BWA picked one randomly. Then, SAMtools^(11)^ and Picard (http://broadinstitute.github.io/picard/) were used to sort the BAM files and do duplicate marking, local realignment, and base quality recalibration to generate the final BAM file for computing the sequence coverage and depth. ANNOVAR (version 2017-07-17, http://annovar.openbioinformatics.org/en/latest/);^(12)^ was performed to annotate the Variant Call Format file obtained in the previous step.

Filter conditions were set to identify the candidate genetic alterations as follows:

1. Remove mutations with ≤ 10× coverage;
2. Remove variant sites in dbSNP and with mutant allele frequency (MAF) > 0.001 in the 1,000 Genomes databases (1,000 Genomes Project Consortium) and the Novo-Zhonghua (in-house unrelated healthy individual database), but include sites with MAF > 0.001 and < 0.1 with COSMIC evidence (http://cancer.sanger.ac.uk/cosmic);^(13-15)^
3. Variations in the exosmic or splicing (10 bp upstream and downstream of splicing sites);
4. Remove synonymous mutations;
5. Retain the nonsynonymous SNVs if the functional predictions using PolyPhen-2, SIFT, MutationTaster, and CADD indicate that SNV is not benign;^(16-19)^

Retain genes identified by Cancer Gene Census (CGC, http://www.sanger.ac.uk/science/data/cancer-gene-census).

***Somatic mutation calling***

Somatic variants were detected using MuTect (version 2)^(20)^ on the exome data of 139 CCAs with paired NATs. To reduce false-positive calls, a panel of NAT was created for each cohort of the same library preparation kit, using all NAT samples in the same cohort. Tumor samples were then used to call somatic mutations against the paired NATs and filtered with the corresponding panel of NATs. To obtain high-quality somatic variants, we further filtered out mutations tending to be false positives. A stringent downstream filter comprising the following criteria was used: retaining only those supported by at least three mutation reads with variant allele frequency (VAF) > 0.08; and VAF < 0.01 in NATs; strand bias 95%; removing mutations in the non-coding regions (such as 30UTR, 50UTR, Intron, and gene intergenic). Somatic insertions and deletions (indels) were called using Strelka27 (v.1.0.17) with default parameters. Accordingly, 26,000 non-silent SCNA calls and 6,000 indel calls were identified for CCA tumors. All somatic mutations identified in these CCA patients were summarized in **Table S2**.

***Significantly mutated gene analysis***

All somatic variants were first annotated by ANNOVAR (version 2017 Jul 17)^(12)^ on RefSeq gene models. To identify significant mutation genes in the CCA cohort, two published methods MutSigCV2 (v.1.41)^(21)^ and OncodriveCLUST (v. 1.1.2)^(22)^ were used to determine significantly mutated genes (SMGs) using the default parameters, and the recurrently mutated genes (≥5 affected cases) detected across our cohort and genes with q values <0.1 (using the recommended significance thresholds) were considered to be significantly mutation. We annotated CCA driver genes with a combination of driver lists: (1) significantly mutated genes in CCA from previous publications;^(2, 23-28)^ (2) a list of drivers from database approaches, including PanCancer (https://www.nature.com/collections/afdejfafdb)*,* OncoKB(https://www.oncokb.org/cancerGenes), COSMIC (https://cancer.sanger.ac.uk/cosmic) and intOGen (https://www.intogen.org/search) sub-setting to only those CCA-related genes; and (3) new potential drivers identified in CCA cohort in this study. 24 genes were selected as potential drivers including 8 SMGs, 16 of which had been reported as recurrent genes in other cohorts. To assess the differences of these driver genes apart from previous cohorts, we included only those with high mutation frequency in each cohort (recurrently mutated genes ≥5 affected cases in EAS and EUR cohorts) and Pearson correlation analysis on the frequency of mutations in CCA-associated genes in seven cohorts (**Fig. 1C**). **Fig. 1D** compared the driver frequencies among cohorts, the p-values were calculated by two-sided Fisher’s exact test, and an FDR cutoff of 0.05 was considered to be statistical significance.

***SCNA calling***

SCNA analysis was performed following SCNA calling pipeline in GATK’s (GATK v 4.1.2.0) Best Practice.^(29)^ GISTIC2 (v.2.0.23)^(30)^ was used to identify the significantly amplified and deleted regions in the CCA cohort. Output segmentations from the GATK pipeline were used as the input for GISTIC2 analysis implemented using GenePattern (https://www.genepattern.org/). Notably, the Seg.CN required by GISTIC was calculated using the depth. ratio estimation from Sequenza as Seg.CN = log_2_ (2 × depth. ratio) – 1. To avoid bias from different sample ploidies, depth. ratios were further divided by the ploidy of each sample to correct for ploidy. The GISTIC parameters were set as follows: -genegistic 1 -smallmem 1 -broad 1 -brlen 0.5 -conf 0.95 -armpeel 1 -savegene 1 -gcm extreme. Chromosome arms were labeled as “altered” in each cohort if GISTIC q < 0.1. Peak regions with FDR < 0.25 were called significant peaks and were annotated with maftools (v.2.8.0). The significant cancer drive genes were annotated in the focal arm regions (**Fig. 2B**).

***SCNA-driven cis- and trans- effects***

SCNAs affecting protein and phosphoprotein abundance in either “*cis*” (within the same aberrant locus) or “*trans*” (remote locus) mode were visualized using “multiOmicsViz” R package.^(31)^ Spearman correlation coefficients and associated multiple-test adjusted p-values were calculated for all SCNA–protein, and SCNA–phosphoprotein pairs, which produced SCNA–protein pairs for 917 genes and SCNA–phosphoprotein pairs for 571 genes.

***Mutation signature analysis***

To uncover mutational processes active in CCA patients, the *de novo* mutational signatures of all 139 tumors with the software of Mutation Signatures in Cancer (MuSiCa).^(32)^ The 96 mutational vectors (or contexts) generated by somatic SNVs based on six base substitutions (C > A, C > G, C > T, T > A, T > C, and T > G) within the 16 possible combinations of neighboring bases for each substitution were used as input data to infer their contributions to observed mutations. MuSiCa using the NMF method as described previously was implemented to decipher the 96×139 matrix by 30 known COSMIC cancer signatures (https://cancer.sanger.ac.uk/signatures/) and infer their exposure contributions. In total, 200 NMF iterations were performed, and each NMF run was iterated until convergence (10,000 iterations without change) or until 1 million iterations were achieved. Four stable and reproducible mutational signatures were deciphered and termed signatures A, B, C, and D. The resulting signature contribution proportions were used to cluster samples using hierarchical clustering with 1-cosine similarity distance and average linkage function using sigminer (v.2.0.5) R package.^(33)^

***Tumor mutational burden (TMB)***

TMB was defined as the number of somatic, coding, base substitution, and indel mutation per megabase genome examined. All base substitutions and indels in the coding region of targeted genes, including synonymous alterations, were also counted to reduce sampling noise, as previously described.^(34)^ While synonymous mutations were not likely to be directly involved in creating immunogenicity, their presence was a signal of mutational processes that would also have resulted in nonsynonymous mutations and neoantigens elsewhere in the genome. Non-coding alterations were not counted. To calculate the TMB per megabase, the total number of mutations counted was divided by the size of the coding region of the targeted territory.

**RNA-seq data analysis**

***RNA-seq reads mapping and normalization***

The quality of RNA-seq raw data from 126 CCAs and 124 paired NATs was assessed using the FastQC (v0.11.9) and the adaptor was trimmed with Trim_Galore (version 0.6.6) before any data filtering criteria were applied. Raw RNA-seq reads were aligned to the human reference genome (GRCh38.p13 assembly) using STAR software (v2.7.7a). The mapped reads were assembled into transcripts or genes using StringTie software (v2.1.4) and the genome annotation file (hg38_ucsc.annotated.gtf). For mRNA expression quantification, the relative abundance of transcripts was then normalized using the FPKM (Fragments Per Kilobase of transcript per Million mapped reads) method. Transcripts with an FPKM score above one were retained, and a total of 10,004 gene IDs were identified in the FU-CCA cohort. All known exons in the annotated file were 100% covered.

***Quality assessment using quality control RNA-seq data***

The filtered RNA-seq data were further evaluated by checking RNA expression abundance distribution (FPKM) followed by log transformation (adding one pseudo-count and log_2_ transformation). A detailed description of the quality evaluation of proteomic data has been given above. Among the 126 paired samples, 124 pairwise samples (tumors and NATs) and 2 tumor samples conformed to a typical unimodal (Gaussian or normal) distribution (dip statistic test), exhibiting high quality. The 2 failed quality assessment samples were excluded from subsequent analyses.

***Transcription factor activity inference***

Transcription factor (TF) activity was inferred using the GSEA algorithm^(35, 36)^ on log_2_ transformed RNA data. The TF targets were collected from DoRothEA^(37)^ and the medium confidence targets (A, B, C) were used for further analysis. The relative activity scores for CCAs and NATs were represented with NES and the p-value was adjusted using the FDR method. TFs with FDR < 0.05 were considered significant.

***Fusion and rearrangement events detection***

Gene fusion and rearrangement events based on 124 paired tumor and NAT RNA-seq data were called using Arriba (version 2.2.1).^(38)^ We refer to the standard workflow produced on the official website (https://github.com/suhrig/arriba), all parameters use the default settings. In order to detect the chimeric reads, STAR (version 2.7.10a) was applied to generate junction files using the latest GRCh37_gencode_v19_CTAT_lib reference genome, with recommended parameter settings to search for chimeric alignments.

**Proteome and phosphoproteome data analysis**

***Data normalization***

Data were normalized using the fraction of total (FOT) method and a relative quantification value defined as protein iBAQ divided by the total iBAQ of all identified proteins in one experiment, was calculated as the normalized abundance of a particular protein or phosphorylation site across all experiments to correct sample loading differences. Finally, the FOT was further multiplied by 1E6 for presentation ease. Then, the FOT (normalized iBAQ intensities) were used in all subsequent quantitative analyses.

***Missing value imputation***

Proteins and phosphosites having > 30% missing data in all samples were excluded before missing value imputation. Missing values (NA) were imputed with 1E–5 to adjust extremely small values for avoiding subsequent algorithm analysis that could not handle missing values.

***Differential expression analysis***

The proteomic data filtered > 30% missing data (n = 6,984 genes) used as input data for differential expression analysis. Then, the protein expression matrix was used to identify proteins differentially expressed in CCAs and NATs using Contrasts functions implemented in the limma (v.3.48.3) R/Bioconductor package. The p-value was adjusted with the Benjamini-Hochberg method and the adjusted p-value cutoff was set to 0.05. A total of 1,274 proteins were identified by differential analysis with fold change > 2 in CCAs compared to NAT.

The parameters for differential analysis of RNA-seq data were as follows: RNA reads data with > 50% FPKM > 1 across all samples (n = 12,465 genes) were used as input data for differential expression analysis. The identification of differential RNA expression between tumors and NATs was implemented using limma R package. The p-values were corrected using the Benjamini-Hochberg method and adjusted p < 0.05 was considered significant. A total of 2,348 genes with fold change > 2 were thus identified using differential analysis.

**Proteomic and phosphoproteomic clustering analysis**

***Proteomic subtyping using NMF***

For proteomic subtyping, the top 3,000 most variable coding proteins (based on median absolute deviation) were chosen from the normalized expression protein matrix (with at least 30% samples showing non-zero expression abundance) among the tumor samples in our CCA cohort. To identify robust clusters, unsupervised clustering of consensus non-negative matrix factorization (cNMF, version 1.1, https://github.com/dylkot/cNMF)^(39)^ was implemented in Python environment (v.3.8.4). Optimal rank parameters were first determined using 50 iterations of range from 2 to 9 (K = 2, 3, 4, 5, 6, 7, and 8) with default settings. To choose the perfect K value for the cNMF clustering algorithm, the trade-off between error and stability of all the run K clusters was plotted to guide K selection (**Fig. S8A**). Based on the plot, we selected a 3-cluster as the best solution for the consensus matrix with K = 3 deemed to be the cleanest separation among clusters. We then performed 300 iterations with the optimal ranks (K = 3) to acquire the final NMF clustering solutions. The heatmap of the consensus matrix showed excellent consistency in the clustering solutions.

***Phosphoproteomic subtyping using CCP***

For phosphoproteomic clustering, we selected the top 1,000 most variable phosphosites (with at least 30% samples showing no missing values) among tumor samples in our cohort (167 tumors). Based on these selected phosphosites, consensus clustering was performed to identify samples with the same phosphorylation characteristics. Consensus clustering was implemented using the R packages ConsensusClusterPlus,^(40, 41)^ and the algorithm parameter settings were: number of repetitions = 1,000 bootstraps; pItem = 0.8 (resampling 80% of any sample); pFeature = 0.8 (resampling 80% of any protein); and the Partitioning Around Medoids (PAM) algorithm were used for clustering within K = 2–6 cluster numbers. The number of clustering was determined by three factors: the average pairwise consensus matrix within consensus clusters, the delta plot of the relative change in the area under the cumulative distribution function (CDF) curve, and the tracking plot for consensus clusters. We selected a 3-cluster as the best solution for the consensus matrix with K = 3 deemed to be the cleanest separation among clusters. Having determined the optimal clustering rank K to achieve robust phosphoproteomic data matrix clusters, we repeated the consensus clustering (ConsensusClusterPlus, CCP) analysis using 200 iterations with random initializations and partitioned the samples into clusters as described above. The clustering analysis of 167 CCA tumors using phosphoprotein abundance (horizontal rows) divided into three phosphoproteomic subtypes (**Fig. S8E**).

***Association of proteomic subtypes and clinical outcomes***

Survival analysis was conducted of CCA patient stratification in different subtypes from NMF clusters. For comparing the survival outcomes among these three subtypes based on proteomic data clustering, the log-rank test was calculated using the R survival package (v.3.2-13) and Kaplan-Meier survival curves were plotted by the R Survminer package (v.0.4.9). To evaluate the prognostic power of the proteomic subtypes, univariable and multivariable Cox analyses were applied with known clinical and pathologic risk factors for CCA progression. All statistical analyses were performed in R (version 4.0.0), and a significance level of 0.05 was used.

**Multi-omics data integrated analysis**

***Differential abundance analysis and pathway enrichment analysis***

Differential analysis of CCA samples with different phenotypes was analyzed using Wilcoxon rank-sum test, including differential proteins and phosphosites carrying mutated versus non-mutated *BAP1*, *AHNAK2*, and 6q14.3 deletion in CCAs and NATs. Differential mRNAs, proteins, and phosphosites in iCCA and eCCA. CCA patients with HBV infection and non-HBV infection groups, as well as across three proteomic subtypes. The p-values were correlated by the BH method and genes with adjusted p < 0.05 and fold change >2 were selected as significant differential proteins and phosphosites. Pathway enrichment analysis of significant genes was performed on the CPDB interaction analysis website (http://cpdb.molgen.mpg.de/) using ORA algorithms. Pathways with an FDR threshold of 0.01 were regarded to be significantly regulated. The proteome and phosphoproteome samples with < 30% missing values were imputed with 10E-5 and used for subsequent analyses except for the differential analysis and pathway enrichment analysis.

***Global heatmap***

Two-way hierarchical clustering was applied to global proteomic data to identify the global differential protein expression and co-expressed protein patterns. Each gene expression value in the global proteomic expression matrix was transformed into a Z-score across all the samples. For the sample-wise and protein-wise clustering, the distance was set as “Euclidean distance’’, and the weight method was ‘‘complete’’. The Z-score-transformed matrix was clustered using the “pheatmap” R package (version 1.0.12).

***Phosphopeptide analysis-kinase and substrate regulation***

KSEA algorithm was used to estimate the kinase activities based on phosphosite abundance. KSEA estimates the changes in kinase activity by measuring and averaging its identified substrate amounts instead of a single substrate, which enhanced the signal-to-noise ratio from inherently noisy phosphoproteomic data.^(42, 43)^ If the same phosphorylation motif was shared by multiple kinases, it was used for estimating the activities of all known kinases. The use of all curated substrate sequences of a particular kinase minimized the overlapping effects from other kinases, thus improving the precise measurement of kinase activities. The information on kinase–substrate relationships was obtained from public databases including PhosphoSite,^(44)^ Phos-pho.ELM,^(45)^ and PhosphoPOINT.^(46)^ The information on substrate motifs was obtained from previous studies^(47)^ or a KSEA dataset using Motif-X.^(42)^

***Kinase activity prediction via PTM-SEA***

Kinase activity scores were inferred from phosphosites by employing phosphosite-specific signature enrichment analysis (PTM-SEA) using the PTM signature database (PTMsigDB) v1.9.0 (https://github.com/broadinstitute/ssGSEA2.0). Sequence windows flanking the phosphorylation sites by 7 amino acids in both directions were used as unique site identifiers. Only fully localized phosphorylation sites as determined through the Spectrum Mill software were considered. Phosphorylation sites on multiple phosphorylated peptides were resolved using the approach described previously,^(48)^ subjecting 29,406 phosphosites to PTM-SEA analysis using the following parameters:

gene.set.database = ‘‘ptm.sig.db.all.flanking.human.v1.9.0.gmt’’

sample.norm.type = ‘‘rank’’

weight = 0.75

statistic = ‘‘area.under.RES’’

output.score.type = ’’NES’’

nperm = 1000

global.fdr = TRUE

min.overlap = 5

correl.type = ‘‘z.score”

***Kinase substrate prediction***

NetworKIN^(49)^ was used to predict kinases for every identified phosphosite in phosphoproteomic data. Substrate sets were generated using the combined set of known substrates from PhosphoSitePlus and UniProt used in Kinase Activity Inference and the predicted substrates from NetworKIN with a NetworKIN score ≥ 5. Kinase Activity Inference was performed as above with the new combined set of substrates.

***Candidate prognostic biomarker analysis for CCA***

The univariate Cox regression model was used to calculate the HR between differentially expressed genes upon the whole proteomic and transcriptomic data to identify biomarkers for CCA prognosis. Based on the median values of protein and RNA abundance, we classified CCA patients into two groups with high and low gene expression. To access CCA robust prognostic biomarkers, we established strict selection criteria as shown below: (1) tumors versus NATs with adjusted p < 0.01 (Wilcoxon rank-sum test) and fold change > 2; (2) The OS log-rank test p < 0.01 and HR > 1.2 or HR < 0.8 in all CCA cohort; (3) The OS analysis was performed separately for iCCA and eCCA, and p < 0.01 and HR > 1.2 or < 0.8 were satisfied for both iCCA and eCCA groups (**Fig. S3L**). All statistical analysis was two-sided and considered p < 0.05 as statistical significance. To further verify the clinical applicability of these 20 proteins, we combined transcriptomic data and found that the mRNA expression of 18 proteins increased in tumors, suggesting their robust features may be used as biomarkers. These 20 proteins were further annotated in conjunction with draggability information from DGIdb,^(50)^ and subcellular localization information from HPA^(51)^ (https://www.proteinatlas.org/) (**Fig. S3M** and **S3N**; **Table S3**).

***Principal components analysis***

To elucidate multi-omic differences between CCAs and matched NATs and reveal molecular alterations upon CCA tumorigenesis, we performed PCAs at all three omic levels (transcriptome, proteome, and phosphoproteome) between tumors and NATs. The PCA function from the “scikit-learn” R package (version 4.0.0)^(52)^ was used for unsupervised clustering analysis with the parameter “n_components = 2” in the expression matrix of global proteomic data containing > 9,000 proteins features. The 95% confidence coverage was represented by a colored ellipse for each group, which was calculated based on the mean and covariance of points in tumors and NATs groups.

***Gene set enrichment analysis (GSEA)***

GSEA pathway enrichment analysis^(36)^ was performed using the clusterProfiler R Package (v.4.0.5)^(53)^ and the functionally annotated pathways were implemented in ggplot2 (v.3.3.5). Gene sets “h.all.v7.2.symbols.gmt”, “c2.cp.reactome.v7.2.symbols.gmt”, and “c2.cp.kegg.v7.2.symbols.gmt” used for GSEA in this study was downloaded from MSigDB database (http://www.gsea-msigdb.org/gsea/downloads.jsp).

***Potential drug target analysis***

To investigate new treatment options for CCA patients, we systematically screened potential drug targets based on multi-omics data. Druggability information of FDA-approved drugs targeting the protein or drugs under clinical trial was based on the Drug Gene Interaction Database (http://www.dgidb.org/). Target proteins that were upregulated in tumors (Benjamini-Hochberg FDR < 0.01, Wilcoxon signed-rank test) with potential curative drugs (antagonist and inhibitor) were considered as potential drug targets. Similar analyses were performed on the phosphoproteomic data to detect tumor-specific drug target phosphosites.

***Protein–protein interaction network construction***

The interaction network among the proteins and phosphoproteins was generated with STRING v 11.0 (https://string-db.org/) using medium confidence (0.4), and experiments and databases as active interaction sources. The network was visualized using Cytoscape version 3.5.1.^(54)^

***Cell cycle analysis***

Multi-gene proliferation scores (MGPS) were calculated from the median-MAD normalized RNA-seq data as described previously.^(55, 56)^ Briefly, MGPS was calculated as the mean expression level of all cell cycle-regulated genes identified in each sample.^(56)^ Apoptosis and E2F TG scores were the ssGSEA normalized enrichment scores from the corresponding MSigDB hallmark gene sets calculated above (pathway projection using ssGSEA).

***Survival analysis***

All the survival analyses presented in this manuscript (such as OS of proteomic and phosphoproteomic subtypes) were based on Kaplan-Meier survival curves (log-rank test).

**Tumor immune microenvironment analysis of CCA tumors**

***Identification of immune clusters based on cell type composition***

The abundance of 64 different cell types in 217 CCAs was computed via xCell.^(57)^ For this analysis, the protein expression matrix, excluding > 30% missing values across all the samples, was utilized. **Table S7** includes the final xCell scores for different cell types for the 217 CCAs. Consensus clustering was performed based on cells only detected in at least 30% of patients (adjusted p < 0.01). This filtering resulted in 29 cell types. To identify sample groups with similar immune/stromal characteristics, consensus clustering was performed using the R packages ConsensusClusterPlus^(41)^ based on the normalized Z-score of these 29 xCell signatures selected above. Specifically, 80% of the original 217 samples were randomly subsampled without replacement and partitioned into six major clusters using the Partitioning Around Medoids (PAM) algorithm, which was repeated 200 times.^(41)^ **Fig. 7A** illustrates the heatmap of scores for key cell types from the 217 CCAs.

***Estimation of stromal and immune scores***

ESTIMATE^(58)^ was also used to infer tumor purity and immune and stromal scores based on RNA-seq and global proteomic data, with default algorithm parameters (**Table S7**). For global proteomic data analysis, only proteins with > 30% missing values across all the samples were utilized. The immune and stromal scores based on global proteomic and RNA-seq data were highly correlated as the Pearson correlation between immune scores based on RNA-seq and proteomic data was > 0.85 and that of stromal scores was > 0.75 for CCA samples (**Table S7**). For this comparison, only samples overlapping between these two data types were considered (i.e., 126 CCA tumors and paired 124 NATs).

***Analysis of immune-related pathways***

To investigate the impact of different biological processes pathway enrichment on immune clusters, the “GSVA” R package was used to conduct GSVA enrichment analysis.^(59)^ For this analysis, the gene set “h.all.v7.2.symbols.gmt”, “c2.cp.reactome.v7.2.symbols.gmt” and “c2.cp.kegg.v7.2.symbols.gmt” for GSVA analysis was downloaded from the MSigDB database (http://www.gsea-msigdb.org/gsea/downloads.jsp). Pathway scores of 217 tumors were computed based on proteomic data with transformed Z-scores. **Table S7** summarizes the pathway scores of different data types. Only the combined Z-scores of some key enriched pathways were included.

***Clinical outcome of immune clusters***

Immune clusters combined with clinical information were utilized to understand the clinical outcome and prognosis survival for different immune groups (**Fig. 7B** and **7C**). Survival analysis was performed to compare OS rate across the six immune clusters survival (3.2-13) R package. Kaplan-Meier curves for OS were generated using the Survminer (0.4.9) R package (**Fig. 7B**).

***Correlation between xCell signatures and clinical features***

Spearman correlation analysis was performed to illustrate associations between xCell signatures and clinical features. Spearman correlation coefficients (r and p) were calculated in R environment (v.4.1.0). r > 0.3 or < −0.3 combined with p < 0.05 were considered strong correlations to investigate the impacts of xCell signatures and clinical features on CCA patient prognosis. The HRs were calculated from the univariate Cox regression model. The log-rank test was used to determine the significance of the differences and p < 0.05 was considered statistically significant.

**Functional experiments**

***Antibodies and chemicals***

Antibodies against TCN1 (Catalog: ab202121), cleaved caspase-3 (Catalog: ab32042), and cleaved PARP (Catalog: ab32064) were purchased from Abcam (Cambridge). The antibody against Actin (Catalog: A00702) was purchased from Genscript (Piscataway). The antibody against Flag (Catalog: M20008) was purchased from Abmart (Berkeley Heights). The Anti-Pan-Suck antibody was used as before.^(60)^

The VB12 concentrations in cultured cells were detected using a human vitamin B12 ELISA kit (Cusabio). Intracellular homocysteine concentrations in cell cultures were measured by a homocysteine-ELISA kit (Bio-Rad) following the manufacturer’s protocol as we used before.^(61)^

***Plasmid constructs and transfection***

Whole-length human TCN1 was amplified from HCCC-9810 cDNA and cloned into the Xho I and EcoR I restriction sites of the pcDNA3.1-Flag/HA vector using CloneExpress MultiS One Step Cloning Kit (Catalog: C113-02, Vazyme). Each plasmid was transfected using Lipofectamine 3000 (Invitrogen, Carlsbad, USA) according to the manufacturer’s instructions.

The primers used were as follows:

TCN1: forward primer, 5’- aacgggccctctagactcgagATGATGAGACAGTCACACCAGCTG-3’, reverse primer, 5’-tagtccagtgtggtggaattcCATGTATTTGCTCCAGCGAACC-3’.

***Small RNA interference***

Synthetic oligos were used for siRNA-mediated TCN1 silencing, and scramble siRNA was used as a control. Cells were transfected with siRNAs using Lipofectamine 3000 (Invitrogen) according to the manufacturer’s protocol. Knockdown efficiency was verified by western blotting. siRNA sequences were as follows: TCN1 (human): 5’-ACUCUGUGAGAAUCAAUGAAACATA-3’.

***Western blot***

Cultured cells or cells extracted from mice tissue and patient-matched peripheral blood mononuclear cells (PBMC) were homogenized with 0.5% NP-40 buffer containing 50 mM Tris-HCl (pH 7.5), 150 mM NaCl, 0.5% Nonidet P-40, and a mixture of protease inhibitors (Sigma-Aldrich). After centrifugation at 12,000 rpm and 4℃ for 15 min, the supernatant was collected for western blotting according to standard procedures. Detection was performed by measuring the chemiluminescence on Typhoon FLA 9500 (GE Healthcare).

***EDU staining***

HeLa cells were cultured at an appropriate concentration for growth and 20 μm EDU was added to the cell culture medium for 1 h. Cells were harvested and washed with PBS twice to remove the remaining medium. Paraformaldehyde (4%) was used to fix the cells at room temperature; 0.5% Triton X-100 in PBS was added and incubated for 20 min at room temperature. The cocktail (PBS: 215 μL, 100mM CuSO4: 10μL, 2mM Azide: 0.6 μL, 1M Sodium Ascorbate: 25 μL) was added for 30 min at room temperature in the dark. DAPI was subsequently added, for nuclear staining. Results were acquired in a flow cytometer or cells were observed under a fluorescence microscope.^(62)^

***Apoptosis analysis***

FITC Annexin V Apoptosis Detection Kit 1 (BD, Biosciences) was used to detect the apoptotic cells. Data were collected on Accuri C6 flow cytometer (BD, Biosciences).

***Cell proliferation assay***

HeLa cell proliferation was tested using CCK-8 kit (Sigma-Aldrich). In brief, 100 μL cell suspension/well was inoculated in triplicate in a 96-well plate. At 0, 24, 48, 72, 96, and 120 h, 10 μl CCK-8 solution was added to each well and incubated at 37 °C for 1 hr. The absorbance at 450 nm was measured using an automatic microplate reader (Synergy4, BioTek).

***Cell viability analysis***

The inhibitory effect of Brivanib, BIBF-1120, and cediranib (purchase from Selleck Chemicals, Houston, TX, USA) on the viability of QBC-939, TFK-1, EGI-1, CCLP1, HuCCT1, and TKKK cells was measured by the CCK-8 assay (Sigma-Aldrich, USA) according to the protocol provided by the manufacturer. Briefly, cells were seeded in 96-well plates (Corning Incorporated, Corning, MA, USA) at a density of ≈5x10^3^ cells/dish in 100 μL of culture media and grown at 37°C for 24 h. Thereafter, they were treated with different concentrations of Brivanib, BIBF-1120, and cediranib for 48h under normoxic or hypoxic conditions, respectively. Subsequently, 10 μL CCK-8 solution was added to each well and the plates were incubated at 37°C for 0-4 h. The optical density of each well was determined at 450 nm with a microplate reader (Bio-Rad, Hercules, CA, USA). All experiments were independently repeated three times. The half-maximal inhibitory concentration (IC50) values of Brivanib, BIBF-1120, and cediranib in QBC-939, TFK-1, EGI-1, CCLP1, HuCCT1, and TKKK cells were calculated using GraphPad Prism 6 software.

***Immunohistochemistry (IHC)***

Formalin-fixed, paraffin-embedded tissue sections of 10 µM thickness were stained in batches for detecting CD34 (marker of vascular endothelial cells), phosphorylation of CDK1 at T161, phosphorylation of MCM2 at S27, and phosphorylation of RB1 at T373 in a central laboratory at the Zhongshan Hospital according to standard automated protocols. Deparaffinization and rehydration were performed, followed by antigen retrieval and antibody staining. CD34 IHC was performed using the Leica BOND-MAX auto staining system (Leica). Rabbit monoclonal anti-CD34 antibody (ab110643, Abcam), phosphorylation of CDK1 at T161(ab201008, Abcam), phosphorylation of MCM2 at S27 (ab109459, Abcam), and phosphorylation of RB1 at T373 (ab52975, Abcam) was introduced, followed by detection with a Bond Polymer Refine Detection DS9800 (Bond). Slides were imaged using an OLYMPUS BX43 microscope (OLYMPUS) and processed using a Scanscope (Leica).

***Metabolite measurement by LC-MS/MS***

The metabolites were extracted using a previously described protocol.^(63)^ Briefly, the samples were resuspended in HPLC-grade H_2_O before flow injecting and analyzing with the 6500 QTRAP mass spectrometer (AB SCIEX) coupled to a Shimadzu HPLC system (LC 20AB) via Multiple reaction monitoring (MRM) mode to only detect the concentration of succinyl-CoA, methionine, and SAM. The relative concentration of Succinyl-CoA, methionine, and SAM could all be analyzed.

***Metabolic enzymes assays***

SDH activity assay was performed by adding 60 µl submitochondrial particles from cells in a 90 mm dish to 140 µl assay buffer containing succinate (0.5–100mM), 0.8mM nitro blue tetrazolium, 0.1 mM phenazine methosulfate, and 0.5mM sodium azide in 100mM phosphate buffer (pH 7.0). The reaction was performed at 25℃, and the production was measured at 574nm using BioTek Epoch Spectrophotometer System. The mitochondrial particles were prepared as described previously.^(64)^ Solubilized complex II from mitochondrial particles was assayed according to the reported protocol.^(65)^

The PDHA1 activity assay was performed in a reaction buffer containing 50 mM KH_2_PO4 (pH 7.0), 1mM MgCl_2_, 2mM sodium pyruvate, 0.2mM thiamin diphosphate, and 0.1 mM 2,6-dichlorophenolindophenol (2,6-DCPIP). The addition of purified PDHA1/PDHB complex initiated the reaction. The reactions were maintained at 30°C. The process of reaction was monitored by measuring 2,6-DCPIP reduction at 600 nm on a Roche spectrophotometer.^(66)^

***In vivo xenograft studies***

Six-week-old Balb/C nude mice were obtained (Shanghai SLAC Laboratory Animal, Shanghai, China) for in vivo xenografts. Control cells and stably overexpressing TCN1 or TCN1 shRNA from HCCC-9810 and QBC-939 cell lines were subcutaneously heterotransplanted into the left and right flank of each mouse. The mice were maintained under conditions as specified. At the end of the experiment, following euthanasia, tumors were excised, weighed, and imaged. All procedures were approved by the Animal Care Committee at Fudan University.

**Statistical methods**

All analyses were performed in R (version 4.1.0) and Python (version 3.8.6) environments. Standard statistical tests were used to analyze the clinical and multi-omics data, including included Fisher’s exact test and Chi-square test for categorical variables, Kruskal-Wallis test and Student's t-test was used to test the statistically significant differences between the subgroups of continuous variables, and Spearman and Pearson correlation was used for continuous variables versus continuous variables. To account for multiple-testing, the *P*-values were adjusted using the Benjamini-Hochberg FDR correction method. Kaplan–Meier estimates overall survival with log-rank tests among strata which are stratified according to the expression of genes (High vs. Low; The median expression values are set as a cut-off), mutational status (*BAP1*-mut vs. *BAP1*-WT, etc.) and predefined subgroups, and the hazard ratio and confidence interval were calculated from Cox proportional hazards regression models. All statistical tests were two-sided, and *P*-values < 0.05 was considered statistical significance.

**Data availability**

Clinical data and supporting stables have been deposited to the National Genomics Data Center (OMIX database: OMIX001128, https://ngdc.cncb.ac.cn/omix/preview/n94CNqN3). The WES data have been uploaded to the National Genomics Data Center (GSA database: HRA002079, https://ngdc.cncb.ac.cn/gsa-human/s/37V9HL94). The RNA-seq data can be viewed in the National Omics Data Encyclopedia (NODE database: OEP002768, http://www.biosino.org/node/review/detail/OEV000280?code=TIPQOBI5). The data of proteomic and phosphoproteomic have been deposited to the ProteomeXchange Consortium (iProX database: IPX0003501000, with subproject ID of IPX0003501001 and IPX0003501002, respectively, https://www.iprox.cn/page/PSV023.html;?url=1649647539240QLBX [PWD: CDOF]). Metabolite data have been uploaded to the National Genomics Data Center (OMIX database: OMIX001103, https://ngdc.cncb.ac.cn/omix/preview/2FVd22gm).

**Supporting Figures Legends**

**Fig. S1. Quality assessments of genomic, transcriptomic, proteomic, and phosphoproteomic data, related to Fig. 1.**

1. Venn diagram summary of the CCA patients in WES, RNA-seq, proteomic, and phosphoproteomic analysis. A total of 217 tumors and 197 NATs were used for proteome profiling (blue circle). A total of 167 tumors and 163 NATs were used for phosphoproteome profiling (red circle). A total of 139 pairs of CCAs and NATs were performed WES (purple circle). A total of 126 tumors and 124 NATs were used for RNA-seq analysis (orange circle).
2. Cumulative number of protein identifications. Orange and blue denote CCA tumors (n = 217), and NATs (n = 197), respectively.
3. Density plot showing the distribution of protein abundances in tumors (orange) and NATs (blue). A unimodal distribution (dip test) is observed. All of the samples pass proteomic quality control.
4. Longitudinal MS quality control using tryptic digest of HEK293T cells. Scatter plots and Spearman correlation coefficients are performed to evaluate the robustness of label-free quantification. Spearman correlation coefficients between repeat experiments with the same samples and the pairwise scatterplot comparison of the different samples, respectively. The average correlation coefficients of the proteome standards were 0.94 (95% CI: 0.93–0.96), exhibiting good reproducibility between these repeat experiments.
5. Spearman correlation coefficients between repeat experiments with the same samples and the pairwise scatterplot comparison of the different samples, respectively. The average correlation coefficients of the phosphoproteome standards were 0.93 (95% CI: 0.90–0.97), exhibiting good reproducibility between these repeat experiments.
6. Scatterplots showing the protein abundance dynamics in tumors (orange) and NATs (blue). Proteins are quantified as a normalized intensity-based absolute quantification (iBAQ) value and log_10_ transformed. The highest and lowest abundance proteins are shown in the box.
7. Distribution of RNA abundances in tumors and NATs by density. Samples that do not pass quality control for transcriptomics were excluded from the subsequent analysis. Unimodal distributions (dip test) are observed.
8. Violin plots showing Signature D (SBS22) exposure in iCCA and eCCA groups (Wilcoxon rank-sum test p < 0.05).
9. Pearson correlation analysis of Signature D (SBS22) exposure and the ImmuneScore. The ImmuneScore is computed via the ESTIMATE algorithm using proteomics data.
10. Overview of the proteomic identifications in CCA. The Venn diagram shows the identification of proteins in tumors and NATs, and the overlap of their shared identified proteins (top panel). Pairwise comparison of proteins is annotated in gray straight lines (bottom). The dashed curves fitted by less regression show the distribution of protein identifications in tumors (orange, n = 217) and NATs (blue, n = 197). See also **Table S1**.
11. Overview of the phosphoproteomic profile of CCA patients. The Venn diagram shows the identification of phosphoproteins and phosphosites in tumors and NATs, and their shared identification numbers. See also **Table S1**.

**Fig. S2. Phosphoproteomic characterization, related to Fig. 1.**

1. MS/MS spectrum of the CDK1 at T161, MCM2 at S27, and RB1 at T373.
2. IHC staining CDK1 at T161, MCM2 at S27, and RB1 at T373 in CCA tumor tissues and NATs. FFPE sections were stained for phosphorylation of CDK1 at T161, MCM2 at S27, and RB1 at T373. The scale bar indicates 20 μm.

**Fig. S3. Identification of significant mutation genes of CCA, related to Fig. 1.**

1. Scatter plot of significantly mutated genes in CCA. Q values (FDR) are calculated by the MutSigCV and OncodriveCLUST algorithms; the red dashed lines on the x and y axes represent mutation counts ≥ 5 and q values < 0.1, respectively.
2. Lollipop plot showing the identified *DPCR1* and *RBM47* mutations in the FU-CCA cohort. Twenty CCA tumors harbor *DPCR1* mutations and 8 CCAs harbor *RBM47* mutations, including missense mutations, insertions, deletions, and frameshift mutations distributed across the entire length of the protein. The colors of the lollipops indicate the type of mutation and numbers represent amino acid positions. The structural domains of the gene are indicated by different color coding as outlined in the Figure.
3. Predicted structure of RBM47 protein in AlphaFold database, highlighting the positions affected by missense mutations with high confidence scores (confidence score of *RBM47-T42M* and *RBM47-A284S* variants are 88. 7 and 94.3, respectively)
4. The mutations of *RBM47* significantly promote cell growth and proliferation in both HCCC-9810 and QBC-939 cells.
5. Western blots on the left and bar plots on the right show the *RBM47* mutations significantly enhanced the expression of P53 compared to RBM47 WT in HCCC-9810 cells.
6. Western blots on the left and bar plots on the right show the *RBM47* mutations significantly enhanced the expression of P53 compared to RBM47 WT in QBC-939 cells.
7. Violin plots showing DPCR1 protein expression in DPCR1-mutant and DPCR1-WT groups (Wilcoxon rank-sum test p < 0.05).
8. The mutations of *DPCR1* significantly promote cell growth and proliferation in both HCCC-9810 and QBC-939 cells.
9. Western blots on the left and bar plots on the right show the *DPCR1* mutations significantly enhanced the phosphorylation of NFKB1A compared to DPCR1 WT in HCCC-9810 cells.
10. Western blots on the left and bar plots on the right show the *DPCR1* mutations significantly enhanced the phosphorylation of NFKB1A compared to DPCR1 WT in QBC-939 cells.

**Fig. S4. Systematic comparison of FGFR2 alterations between cohorts and portray the downstream biological events associated with FGFR2 alterations, related to Fig. 1.**

1. Stacked plots showing the distribution of the *FGFR2* fusions in iCCA (n = 83) and eCCA (n = 41), the different color blocks indicate the fusions (dark blue) and WT (cyan) groups, and the numbers represent the proportion of affected samples (left panel), and the right panel depicts the comparison of *FGFR2* fusions frequency between our iCCA cohort (n = 83) and Dong iCCA cohort (n = 253; Two-sided Fisher’s exact test).
2. Venn diagram showing the overlap of *FGFR2* rearrangement partners in our iCCA cohort and Dong cohort.
3. Barplot showing the distribution of *FGFR2* rearrangement partners throughout the genome position.
4. Lollipop plot showing *FGFR2* mutations identified in the FU-CCA cohort. Six CCA tumors harbor *FGFR2* mutations, including all six missense mutations distributed across the entire length of the protein. The numbers of the lollipops represent the amino acid positions, and the structural domains of the gene are indicated by different color coding as outlined in the figure.
5. Violin plots showing FGFR2 expression of mRNA and protein levels in *FGFR2*-altered and FGFR2-WT groups (Wilcoxon rank-sum test p < 0.05).
6. Barplots showing the enrichment of specific pathways in *FGFR2* alterations vs. WT groups, based on the upregulated genes. The colors of the barplots indicate the impacted pathways based on transcriptome (dark blue) and proteome (cyan) levels.
7. Scatterplots showing significantly upregulated RNA and cognate protein expressions in CCA patients with and without *FGFR2* alterations (left panel), and the right panel depicts the significantly upregulated phosphosites and their cognate protein expressions in *FGFR2* alterations vs. WT groups. The colors of the dots indicate the impacted pathways based on upregulated genes on transcriptome, proteome, or phosphoproteome levels.
8. Systematic diagram illustrating significantly enriched downstream pathways associated with *FGFR2* alterations.

**Fig. S5. The identification of FGFR2 fusions and mutations in the FU-CCA cohort, related to Fig. 1.**

1. Stacked plots showing the distribution of the *FGFR2* mutations in iCCA (n = 102) and eCCA (n = 37), the p-value was using two-sided Fisher’s exact tests.
2. Violin plots depicting FGFR2 expressions of mRNA and protein levels in *FGFR2*-fusion and FGFR2-WT groups (Wilcoxon rank-sum test p < 0.05).
3. Violin plots depicting FGFR2 expressions of mRNA and protein levels in *FGFR2*-mutant and FGFR2-WT groups (Wilcoxon rank-sum test p < 0.05).
4. Differential expression of seven *FGFR2*-related binding FGF ligands in CCA patients with and without *FGFR2* alterations (Wilcoxon rank-sum test).

**Fig. S6. Profiles of SCNAs in CCA, related to Fig. 2.**

1. Correlations of SCNA (x-axes) to RNA (left) and protein (right) expression (y-axes) with SCNA *cis-* and *trans-*effects. Significant (FDR < 0.05) positive and negative correlations are indicated in red and green, respectively. Genes are ordered by chromosomal locations on the x and y axes. The red diagonal line indicates CNA-driven *cis*-effects. Trans-effects appear as vertical red and green lines.
2. Heatmap revealing the 24 CAGs correlations between SCNAs and their cognate gene products in the FU-CCA and Dong cohorts (mRNA expression [up], protein expression [bottom]; top panel in each module) in the Dong cohort. Bottom heatmap represents the impacts of SCNA, mRNA, and protein level alterations on CCA patients’ OS. Survival Kaplan-Meier analysis is performed and log-rank tests are employed to identify significant differences. HRs are calculated using the univariate Cox regression model. The asterisks represent the statistical p-values (*p < 0.05; **p < 0.01; ***p < 0.001).
3. Cox regression analysis of significant arm-level and focal arm-level SCNA events.
4. Boxplots showing SH3BGRL2 expression of mRNA and protein levels in *SH3BGRL2*-deletion and SH3BGRL2-WT groups (Wilcoxon rank-sum test p < 0.05).
5. Survival Kaplan-Meier analysis of patients stratified by SH3BFRL2 median RNA and protein expressions. The numbers in parentheses represent the sample sizes of the involved groups. P-values are calculated using the two-sided log-rank test.
6. Correlations of SH3BGRL2 protein expression with some pathways, of which the significant ones are labeled in red (Spearman correlation p < 0.05).
7. Correlations of the *O*-linked glycan biosynthesis pathway with other pathways, of which the significant ones are labeled in red (Spearman correlation p < 0.05).

**Fig. S7. The alterations of multi-omic profiles in CCA tumors and candidate prognosis biomarker identification, related to Fig. 3.**

1. PCA plots of proteomic expression in tumors (orange) and NATs (blue).
2. Heatmap showing the enriched pathways of significantly differentially expressed proteins (Benjamini-Hochberg adjusted p < 0.05, Wilcoxon rank-sum test) among tumors and NATs from iCCA and eCCA.
3. PCA plots showing RNA abundance in CCA tumors (orange triangles) and NATs (blue circles).
4. Two-sided bar graphs showing protein-specific pathway enrichment in CCA tumors (orange bars) and NATs (blue bars), based on the Hallmark genesets.
5. PCA plots showing phosphoprotein abundance in CCA tumors (orange triangles) and NATs (light blue circles).
6. Two-sided bar graphs showing phosphoprotein-specific pathway enrichment in tumors (orange bars) and NATs (light blue bars), based on the Hallmark genesets.
7. Scatterplots depicting the changes of protein (x-axis) and RNA (y-axis) in tumors compared to those in NATs. Linear regression of all mRNA-protein pairs (blue line) is shown (Spearman correlation r = 0.58, p < 2.2E–16). Dot colors indicate the shared or site-specific elevations.
8. Scatterplots depicting the differences in protein (x-axis) and phosphosite (y-axis) abundance in tumors and NATs. The blue line indicates linear regression of all protein-phosphosite pairs (Spearman correlation r = 0.63, p < 2.2E–16). Red points indicate the phosphosites with a fold change > 2.
9. KSEA of kinase activities in tumors (red) and NATs (blue).
10. Boxplots showing the significantly elevated phosphosite abundance in tumors (yellow) compared to NATs (red; Wilcoxon rank-sum test).
11. Boxplots indicating CDK (CDK1/2) and MAPK (MAPK1/3) protein expression in tumors (yellow) and NATs (red; Wilcoxon rank-sum test).
12. Survival Kaplan-Meier curves of TCN1 mRNA expressions in the FU-CCA cohort.
13. Survival Kaplan-Meier curves of TCN1 mRNA expression in the CHOL cohort from TCGA datasets (log-rank test). The median expression values are used as a cutoff.
14. Survival Kaplan-Meier curves of TCN1 mRNA and protein expression in the Dong cohort (log-rank test). The median expression values are used as a cutoff.

**Fig. S8. Consensus clustering of proteomic and phosphoproteomic profiles in CCA cohort, related to Fig. 4.**

1. Schematic of the consensus matrix factorization pipeline.
2. Overview of significantly enriched pathways across the three proteomic subtypes. Average protein abundances are indicated in each proteomic subtype.
3. Stacked barplots illustrating significant clinical feature distribution across the proteomic subtype. The clinical feature indicators are annotated on the right graph panel. The p-value is calculated using Fisher’s exact test.
4. Sankey plot revealing the association between our proteomic subtypes characteristics and subgroups reported by Dong et al.’s studies.
5. Identification of clusters based on phosphoproteomic data of the FU-CCA cohort (n = 167) using ConsensusClusterPlus R package upon their abundance (**Supporting Materials and Methods**). K was tested from 2 to 6 and consensus clustering was based on 1,000 resampled datasets. Consensus matrices, as well as consensus cumulative distribution function (CDF) plot, delta area (change in CDF area) plot, and tracking plot plots are shown.
6. ROS accumulation in *BAP1*-mutant and BAP1-WT groups (Wilcoxon rank-sum test).
7. Boxplots illustrating the downregulation of SOD3 protein expression in tumors and NATs (Wilcoxon rank-sum test).
8. Survival Kaplan-Meier curves of BAP1 mRNA expression in the CHOL cohort from TCGA datasets (log-rank test). The median expression values are used as a cutoff.
9. Boxplots showing PARP1 mRNA and protein expressions in *BAP1*-mutant and BAP1-WT groups (Wilcoxon rank-sum test p < 0.05).
10. Survival Kaplan-Meier curves of PARP1 protein expression (log-rank test).
11. Spearman correlation analysis of significant enrichment pathways with PARP1 protein abundance.
12. Boxplots showing the elevation of GAPDH protein expression in *BAP1*-mutant and BAP1-WT groups (Wilcoxon rank-sum test p < 0.05).

**Fig. S9. Systematic comparison of CCA significant mutations events driven by *ARID1A* and *BAP1*, related to Fig. 4.**

1. Pie charts depicting the proportion of patients with *BAP1* and *ARID1A* mutation events. The bar chart depicts the number of samples affected by each mutation type.
2. Kaplan-Meier curves for overall survival based on the *ARID1A* mutation (left panel) and the right panel characterized by WT (red), *BAP1* mutation (orange) *ARID1A* mutation (blue), and co-mutations (purple) altered samples in the FU-CCA cohort, the p-value is calculated using the log-rank test.
3. Stacked plots representing the distribution of the *BAP1* and *ARID1A* mutations across anatomic subtypes. The color blocks indicate the alternated types and numbers represent the number of alterations. The p-value is calculated from the two-sided Fisher’s exact test and a cutoff of 0.05 is considered a significant difference.
4. Stacked plots representing the distribution of the *BAP1* and *ARID1A* mutations across proteome subtypes. The color blocks indicate the alternated types and numbers represent the number of alterations. The p-value is calculated from the two-sided Fisher’s exact test and a cutoff of 0.05 is considered a significant difference.
5. Violin plots showing ARID1A expressions of mRNA and protein levels in *ARID1A*-mutant and ARID1A-WT groups (Wilcoxon rank-sum test p < 0.05).
6. Bar charts showing the enrichment of specific pathways in *ARID1A* mutations and WT, based on the upregulated genes. The colors of the bar plots indicate the influenced pathways based on transcriptome (dark blue) and proteome (cyan) levels.
7. Bar charts showing the enrichment of specific pathways in *ARID1A* mutations and WT, based on the downregulated genes. The colors of the bar plots indicate the influenced pathways based on transcriptome (dark blue) and proteome (cyan) levels.
8. Volcano plots showing the effect of *ARID1A* mutation on the gene expression of RNA and protein levels.

**Fig. S10. Reproduced our HBV findings in the other published datasets with non-HBV data, related to Fig. 5.**

1. Overall survival analysis of CCA patients with and without HBV infection (log-rank test). The CCA patients include the non-HBV cases from published datasets.
2. Volcano plot showing p-value versus HR of different focal peaks. The 6p22.2 amplification is labeled in red for its influence on good prognosis.
3. Frequency of 6p22.2 amplification peak in CCA patients with and without HBV infection.
4. Boxplots showing HBV positives have higher levels of CD4+ T cells based on xCell analysis (Wilcoxon rank-sum test).
5. Overall survival analysis of CCA patients with the high and low levels of CD4+ T cells (log-rank test).

**Fig. S11. Characterization of subgroups of iCCA and eCCA, related to Fig. 6.**

1. Genomic characteristics of some mutated genes and focal events in iCCA and eCCA. The frequency is indicated on the right of the bar chart. The significance is labeled on the left of the bar chart.
2. Significantly altered genes of iCCA and eCCA patients, with a zoom-in of some genes for more details.
3. PCA of global proteomic data showing clear separation of iCCAs and eCCAs.
4. Proteomic pathway enrichment revealing significantly enriched pathways of iCCA and eCCA.
5. PCA of global phosphoproteomic data showing clear separation of iCCAs and eCCAs.
6. Phosphoproteomic pathway enrichment revealing significantly enriched pathways of iCCA and eCCA.
7. Volcano plot showing the p-values versus correlation for cis-effects of SCNA−protein upregulating in eCCA. Four cis SCNA−protein cascade proteins (F11, FGA, FGB, and FGG) belong to the coagulation pathway.
8. Average protein abundances of FGA, FGB, and FGG are significantly correlated with the GSEA score of the VEGF signaling pathway (Spearman correlation r = 0.5, p < 2.2E-16).
9. Heatmap of average protein abundances of FGA, FGB, and FGG with proteins from the VEGF signaling pathway. The Spearman correlation coefficient is calculated with p-values and displayed in log 10 scale.
10. Drug responses to VEGF inhibitors in eCCA cell line are more sensitive than those in iCCA cell lines (Mann-Whitney U test p = 0.0186 and 0.0194, respectively).
11. Dose-response curves (left panel) and half-maximal inhibitory concentration (IC50) values (right panel) of Brivanib we determined on day 2 after inhibitors adding. The data represent the mean values ± SD (n =3).
12. Dose-response curves (left panel) and half-maximal inhibitory concentration (IC50) values (right panel) of Cediranib we determined on day 2 after inhibitors adding. The data represent the mean values ± SD (n =3).
13. Immunohistochemistry of CD34 protein expression in eCCA (upper panel) iCCA (lower panel) samples.

**Fig. S12. Systematic analysis of the genotype associations with large vs small duct subtypes, related to Fig. 6.**

1. Venn diagram summarizing the significantly mutated gene lists correlated with large and small bile duct (BD). The p-value is calculated from the two-sided Fisher’s exact test and a cutoff of 0.05 is considered a significant difference. The cancer-associated genes are output from multiple public cancer datasets.
2. Stacked plots representing the distribution of the different mutation types in large iBD (n = 12) and small iBD (n = 76), the different color blocks indicate the mutations (dark blue) and WT (cyan) group, and the numbers represent the proportion of affected samples (two-sided Fisher’s exact test).
3. Violin plots showing SMAD4 expression of mRNA and protein levels in *SMAD4*-mutant and SMAD4-WT groups in the FU-CCA cohort (Wilcoxon rank-sum test p < 0.05).
4. Violin plots showing SMAD4 expression of mRNA and protein levels in *SMAD4*-mutant and SMAD4-WT groups in the Dong iCCA cohort (Wilcoxon rank-sum test p < 0.05).
5. Bar chart showing the enrichment of specific pathways in *SMAD4* mutations vs. WT, based on the upregulated genes. The colors of the bar plots indicate the impacted pathways based on transcriptome (dark blue) and proteome (cyan) levels.
6. Violin plots showing clinical CA19-9 level in *SMAD4*-mutant and SMAD4-WT groups (Wilcoxon rank-sum test p < 0.05).
7. Bar chart showing the enrichment of specific pathways in *SMAD4* mutations vs. WT, based on downregulated genes. The colors of the bar plots indicate the impacted pathways based on transcriptome (dark blue) and proteome (cyan) levels.
8. Heatmap indicating the expression of selected target genes (TGs) of the SMAD4 transcription factor among the *SMAD4*-mutant and SMAD4-WT groups, and the pathways enrichment of these TGs is annotated on the left panel (Wilcoxon rank-sum test p < 0.05).
9. Stacked plots showing the distribution of the 6q deletion and 6q14.3 deletion in large iBD (n = 12) and small iBD (n = 76), respectively.
10. Survival Kaplan-Meier curves of iCCA patients with 6q14.3 deletion (blue) and wild-type (orange) in iCCA patients of the Fudan-CCA cohort (two-sided log-rank test p < 0.05). HR, hazard ratio; MST, Median Survival Time (months). HRs are calculated using a univariate Cox regression model.
11. Violin plots showing the protein expression of SH3BGRL2 on 6q14.3 in the large iBD and small iBD groups (Wilcoxon rank-sum test p < 0.05).

**Fig. S13. Immune infiltrations in CCAs, related to Fig. 7.**

1. Identification of immune clusters (K = 2–6) based on proteomic data of Fudan CCA cohort (n = 217) by consensus clustering upon their abundance (STAR Methods). Consensus matrix, consensus cumulative distribution function (CDF) plot, delta area (change in CDF area) plot, and tracking plot plots are shown.
2. Pearson correlation analysis of immune checkpoint molecules.
3. Pearson correlation analysis across xCell signatures.

**Fig. S14. TCN1 promotes tumor cell growth through enhancing VB12 metabolism, related to Fig. 8.**

1. Low expression of TCN1 in normal bile duct tissue, from GEPIA database.
2. Increased DNA synthesis after TCN1 overexpression was observed through 5-ethynyl-2’-dexyurdine (EDU) staining in both HCCC-9810 and QBC-939 cell lines.
3. Increased DNA synthesis after VB12 supplementation was observed through 5-ethynyl-2’-dexyurdine (EDU) staining in both HCCC-9810 and QBC-939 cell lines.
4. Utilizing 5-ethynyl-2’-dexyurdine (EDU) staining indicated that TCN1 knock down decreased DNA synthesis in both HCCC-9810 and QBC-939.
5. TCN1 overexpression in cells or VB12 supplementation in the cultured medium cannot significantly promote growth and proliferation in a gastric epithelial cell line (GES1) and human non-small cell lung cancer cell line (HCC827).
6. VB12 involving in methionine and succinyl-CoA metabolism pathways.
7. Decreased succinyl-CoA and pan-lysine-succinylation levels by TCN1 knockdown.

**Supporting Tables**

**Table S1. Clinicopathologic information and multi-omic data in CCA cohort, related to Fig. 1.**

1. Clinical_information. Clinicopathologic information of 217 CCA patients.
2. Drive genes & signatures. This sheet contains information on 24 CCA driver mutation genes and mutation signatures.
3. WES calling mutations. This sheet contains information of 23,560 non-silent single-nucleotide variants (SNVs) and 6,051 small insertions-deletions (INDELs) identified by WES in 139 CCAs.
4. CNAs. Significant amplification and deletion of copy number in CCAs.
5. RNA_seq (FPKM). This sheet contains information on 12,724 mRNA at least quantified in more than 50% across 124 pairs of tumor and NAT samples. The values were subjected to normalize the data using the FPKM method and log2 transformation.
6. Protein_profiling. This sheet contains information on 6,875 proteins at least quantified in more than 30% across all 217 tumor samples. The values were subjected to FOT normalization by column to correct the protein profiling data.
7. Phosphosite_ profiling. Phosphosites abundance quantified at least 30% samples across all phosphoproteome-based samples.
8. Phosphoprotein_abudance. This sheet contains information on 9,109 phosphoproteins quantified in samples. The values were subjected to normalize the data using the FOT method.
9. Protein identifications. This sheet contains information on the number of protein identifications for each sample at the protein level.
10. HEK293T quality control. For the quality control of the performance of mass spectrometry, the HEK293T cell lysate was measured every three days as the quality-control standard in proteome and phosphoproteome, respectively.
11. FGFR2 & kinase fusions. This sheet contains *FGFR2* and other kinase gene fusions based on RNA_Seq data.
12. Comparison of demographic and clinicopathological characteristics with multiple cohorts.
13. Comparison of demographic and clinicopathological characteristics with the Dong cohort.

**Table S2. Impacts of SCNAs on RNA, protein, and phosphoprotein expression of CCA cohort, related to Fig. 2.**

1. GISTIC2 (arm). Copy number alteration in arm level.
2. GISTIC2 (focal). Copy number alteration information (amplification and deletion) of focal arm event in cytobands.
3. CNV_on_RNA_cor_*cis*. The list of 3,031 SCNAs with *cis*-effects on RNA level.
4. CNV_on_protein_cor_*cis*. The list of 919 SCNAs with *cis*-effects on RNA level.
5. CNV_on_phosphoprotein_cor_*cis*. The list of 570 SCNAs with *cis*-effects on RNA level.
6. Three_multiomics_*cis*_merge. Overlap of *cis*-effects observed at RNA, protein, and phosphoprotein levels (FDR < 0.05).
7. Multiomic_pathway_enrichment. Significant pathway enrichments based on significant *cis*-events observed at RNA, protein, and phosphoprotein levels.
8. 24_CAGs_*cis*. Overlap of cancer-associated genes (CAGs) with significant *cis*-effects observed at RNA, protein, and phosphoprotein levels (FDR < 0.05).
9. CCA_arm_OS. Cox regression analysis of significant arm-level SCNA events. p < 0.05 in the two-sided log-rank test was considered statistically significant.
10. Focal_arm_OS. Cox regression analysis of significant focal arm-level SCNA events. p < 0.05 in the two-sided log-rank test was considered statistically significant.
11. 6q14.3_*cis*_protein_OS. Survival analysis of the protein expression in the 6q14.3 focal arm. The results reveal only the SH3BGRL2 exhibit significant association (log-rank test).
12. SH3BGRL2_associated_pathway. Spearman correlation analysis of SH3BGRL2 protein abundance and the GSVA score of pathways.
13. SH3BGRL2_other_cancers. Survival analysis of SH3BGRL2 RNA expression in other cancer cohorts (log-rank test).
14. Glycan_pathway_molecular_OS. Survival analysis of the protein expression of glycan pathway-associated molecules. Hazard ratios are calculated using a univariate Cox regression model.
15. GLYCAN_molecular_cor. Spearman correlation analysis of the *o*-linked glycan biosynthesis pathway and the EMT-related protein abundance. P-values < 0.05 and Spearman correlation > 0.3 considered significant.

**Table S3. The alterations of transcriptomic, proteomic, and phosphoproteomic profiles in CCAs compared to NATs, related to Fig. 3.**

1. GSEA_results (T-NAT). Significantly differentially regulated pathways between CCAs and NATs (FDR < 0.05, NES > 1.5 and NES < -1.5).
2. CCAs_NATs_heatmap. This sheet contains enriched pathways of significantly differentially expressed proteins between CCAs and NATs (Benjamini-Hochberg adjusted p < 0.05, Wilcoxon rank-sum test).
3. RNA_protein_cor_scatter. Spearman correlation between the changes of protein and RNA in CCAs compared to NATs.
4. Pathway_enrichment. Pathway enrichment of the elevations of shared or site-specific genes at the mRNA and protein levels.
5. TF_activity. Transcription factors (TFs) activity inference from transcriptomic data between CCAs and NATs.
6. TF-TG_network. This sheet contains information on the transcription factors (TFs)-target gene (TGs) network diagram.
7. Kinase_activity. kinases activity inferred from phosphorylation of its substrates (normalized enrichment score).
8. Kinase_substract_network. This sheet contains information on kinase activity and corresponding phosphorylated substrates abundance between CCAs and NATs.
9. Kinase_substract_phosphosite. Analysis of the changes in phosphorylation sites in tumors compared to NATs.
10. Vitamin_pathway. Significantly differential proteins are involved in Vitamin metabolic pathways (Benjamini-Hochberg adjusted p < 0.05). The specific families of vitamin metabolism in which these molecules are annotated.
11. RNA_TN_del50%_TN_diff. The list of 8,917 genes with significant differences at the transcriptome level (FDR < 0.05). We first filtered this dataset at least 50% of samples with non-missing values for differential gene analysis.
12. RNA_TN_del50%_TN_diff. The list of 5,503 genes with significant differences at the proteome level (FDR < 0.05). We first filtered this dataset at least 30% of samples with non-missing values for differential gene analysis.
13. Pathway_enrichments. The enrichment of specific pathways based on the screened significantly different genes in mRNA and protein level, respectively.
14. CCA_biomaker_candidate. This sheet contains the information of the screened 40 candidate prognosis proteins.

**Table S4. Proteomic subtypes of CCA and their associations with clinical outcomes, related to Fig. 4.**

- 1. NMF_subtype_top_molecular. This sheet contains molecules with high weights in each NMF subtype.
  2. Mutation & CNAs. Mutation and CNAs across the three proteomics subtypes.
  3. Subtype_pathway_enrichment. Pathway enriched by proteins significantly upregulated in three proteomics subtypes.
  4. Phosphoprotein_CCP_cluster. The identification of three clusters based on phosphoproteomic data (n = 167) using ConsensusClusterPlus R package.
  5. TME_and_Mutation_signature. xCell signatures and mutation signatures across proteomic subtypes.
  6. Subtype_kinase_activty. Kinase activity across proteomic subtypes.
  7. Kinase_substract_network. This sheet contains information on kinase activity and corresponding phosphorylated substrates abundance in three proteomic subtypes.
  8. Subtype_*BAP1*_mutation. This sheet contains information on *BAP1* mutation across proteomic subtypes.
  9. BAP1_GSEA_analysis. GSEA result of pathway enrichment in *BAP1* mutation versus WT groups.
  10. Pathway_protein_OS. Survival analysis of significant proteins in TCA cycle and fatty acid metabolism pathways. Hazard ratios (HR) are calculated using univariate Cox regression model (log-rank test p-value < 0.05).
  11. PARP1_pro_cor_pathway. Spearman correlation analysis of significant enrichment pathway with PARP1 protein abundance.
  12. PARP1_cor_molecular. This sheet contains the information of molecules of significant pathways correlated with PARP1 protein abundance.

**Table S5. The impact of HBV infection on CCA patients, related to Fig. 5.**

- 1. HBV infection_heatmap.
  2. Multi-omic profile of LRRC16A.

**Table S6. Differentially expressed proteins and phosphoproteins between Large iBD and Small iBD, related to Fig. 6.**

- 1. iCCA_eCCA_heatmap.
  2. CNV_Pro_F11_FGA_FGB_FGG.
  3. FGA_B_G and VEGF pathway.
  4. Large iBD_Small iBD_diff. The list of 832 differentially expressed (DE) proteins. The 832 proteins that are differentially expressed in Large iBD and Small iBD. Log2 fold-change (FC) represents log2 (ratio of Large iBD to Small iBD).
  5. Pathway_enrichment. Pathway enriched by proteins significantly upregulated in in large iBD and small iBD tissues.
  6. Large iBD_Small iBD_pathways.

**Table S7. Immune clusters and immune infiltrations of CCAs, related to Fig. 7.**

- 1. xCell signature & Immune Cluster. This sheet contains information on immune/stromal signatures deconvoluted using xCell algorithm based on proteomic data in 217 CCAs.
  2. Clinical_xCell_score_cor. Spearman correlation analysis of xCell signatures with clinical features.
  3. Estimate_score. Estimate score of immune-cell-specific contributions to each tumor, protein data was analyzed using ESTIMATE (R package).
  4. RNA_immune_ICI_target. Expression of immune checkpoint molecules across the six immune clusters at the RNA level.
  5. Clinical_and_xCellSig_OS. Survival analysis of clinical features and xCell signatures, HRs are calculated from univariate Cox regression model. P-values < 0.05 considered to be statistically significant.
  6. Neutrophils_cor_focal_arm. Spearman correlation analysis of focal arm alterations with neutrophil infiltrations. Spearman correlation p-value < 0.05 and r > 0.2 considered as statistically significant.
  7. CA19-9_cor_focal_arm. Spearman correlation analysis of focal arm alterations with serum CA19-9 levels. Spearman correlation p-value < 0.05 and r > 0.2 considered as statistically significant.
  8. 1p36.33 *cis*-effect on RNA_pro. This sheet contains information on CNVs in 1p36.33 focal with significant *cis*-effect on RNA and protein levels, respectively.

**Table S8. Target-metabolic data, related to Fig. 8.**

1. Target-metabolic data of TCN1 overexpressed cell line with control.

**REFERENCES**

1. Zou S, Li J, Zhou H, Frech C, Jiang X, Chu JS, Zhao X, et al. Mutational landscape of intrahepatic cholangiocarcinoma. Nat Commun 2014;5:5696.

2. Jusakul A, Cutcutache I, Yong CH, Lim JQ, Huang MN, Padmanabhan N, Nellore V, et al. Whole-Genome and Epigenomic Landscapes of Etiologically Distinct Subtypes of Cholangiocarcinoma. Cancer Discov 2017;7:1116-1135.

3. Dong L, Lu D, Chen R, Lin Y, Zhu H, Zhang Z, Cai S, et al. Proteogenomic characterization identifies clinically relevant subgroups of intrahepatic cholangiocarcinoma. Cancer Cell 2022;40:70-87 e15.

4. Reisfeld B, Mayeno AN. What is computational toxicology? Methods Mol Biol 2012;929:3-7.

5. Feng J, Ding C, Qiu N, Ni X, Zhan D, Liu W, Xia X, et al. Firmiana: towards a one-stop proteomic cloud platform for data processing and analysis. Nat Biotechnol 2017;35:409-412.

6. Schwanhausser B, Busse D, Li N, Dittmar G, Schuchhardt J, Wolf J, Chen W, et al. Global quantification of mammalian gene expression control. Nature 2011;473:337-342.

7. Zhang W, Zhang J, Xu C, Li N, Liu H, Ma J, Zhu Y, et al. LFQuant: a label-free fast quantitative analysis tool for high-resolution LC-MS/MS proteomics data. Proteomics 2012;12:3475-3484.

8. Taus T, Kocher T, Pichler P, Paschke C, Schmidt A, Henrich C, Mechtler K. Universal and confident phosphorylation site localization using phosphoRS. J Proteome Res 2011;10:5354-5362.

9. Li H, Durbin R. Fast and accurate short read alignment with Burrows-Wheeler transform. Bioinformatics 2009;25:1754-1760.

10. Kent WJ, Sugnet CW, Furey TS, Roskin KM, Pringle TH, Zahler AM, Haussler D. The human genome browser at UCSC. Genome Res 2002;12:996-1006.

11. Li H, Handsaker B, Wysoker A, Fennell T, Ruan J, Homer N, Marth G, et al. The Sequence Alignment/Map format and SAMtools. Bioinformatics 2009;25:2078-2079.

12. Wang K, Li M, Hakonarson H. ANNOVAR: functional annotation of genetic variants from high-throughput sequencing data. Nucleic Acids Res 2010;38:e164.

13. Genomes Project C, Abecasis GR, Auton A, Brooks LD, DePristo MA, Durbin RM, Handsaker RE, et al. An integrated map of genetic variation from 1,092 human genomes. Nature 2012;491:56-65.

14. Sherry ST, Ward MH, Kholodov M, Baker J, Phan L, Smigielski EM, Sirotkin K. dbSNP: the NCBI database of genetic variation. Nucleic Acids Res 2001;29:308-311.

15. Xia H, Xu H, Deng X, Yuan L, Xiong W, Yang Z, Deng H. Compound heterozygous GJB2 mutations associated to a consanguineous Han family with autosomal recessive non-syndromic hearing loss. Acta Otolaryngol 2016;136:782-785.

16. Adzhubei I, Jordan DM, Sunyaev SR. Predicting functional effect of human missense mutations using PolyPhen-2. Curr Protoc Hum Genet 2013;Chapter 7:Unit7 20.

17. Kircher M, Witten DM, Jain P, O'Roak BJ, Cooper GM, Shendure J. A general framework for estimating the relative pathogenicity of human genetic variants. Nat Genet 2014;46:310-315.

18. Ng PC, Henikoff S. SIFT: Predicting amino acid changes that affect protein function. Nucleic Acids Res 2003;31:3812-3814.

19. Schwarz JM, Rodelsperger C, Schuelke M, Seelow D. MutationTaster evaluates disease-causing potential of sequence alterations. Nat Methods 2010;7:575-576.

20. Cibulskis K, Lawrence MS, Carter SL, Sivachenko A, Jaffe D, Sougnez C, Gabriel S, et al. Sensitive detection of somatic point mutations in impure and heterogeneous cancer samples. Nat Biotechnol 2013;31:213-219.

21. Lawrence MS, Stojanov P, Polak P, Kryukov GV, Cibulskis K, Sivachenko A, Carter SL, et al. Mutational heterogeneity in cancer and the search for new cancer-associated genes. Nature 2013;499:214-218.

22. Tamborero D, Gonzalez-Perez A, Lopez-Bigas N. OncodriveCLUST: exploiting the positional clustering of somatic mutations to identify cancer genes. Bioinformatics 2013;29:2238-2244.

23. Moeini A, Sia D, Zhang Z, Camprecios G, Stueck A, Dong H, Montal R, et al. Mixed hepatocellular cholangiocarcinoma tumors: Cholangiolocellular carcinoma is a distinct molecular entity. J Hepatol 2017;66:952-961.

24. Nakamura H, Arai Y, Totoki Y, Shirota T, Elzawahry A, Kato M, Hama N, et al. Genomic spectra of biliary tract cancer. Nat Genet 2015;47:1003-1010.

25. Farshidfar F, Zheng S, Gingras MC, Newton Y, Shih J, Robertson AG, Hinoue T, et al. Integrative Genomic Analysis of Cholangiocarcinoma Identifies Distinct IDH-Mutant Molecular Profiles. Cell Rep 2017;18:2780-2794.

26. Wardell CP, Fujita M, Yamada T, Simbolo M, Fassan M, Karlic R, Polak P, et al. Genomic characterization of biliary tract cancers identifies driver genes and predisposing mutations. J Hepatol 2018;68:959-969.

27. Goeppert B, Toth R, Singer S, Albrecht T, Lipka DB, Lutsik P, Brocks D, et al. Integrative Analysis Defines Distinct Prognostic Subgroups of Intrahepatic Cholangiocarcinoma. Hepatology 2019;69:2091-2106.

28. Montal R, Sia D, Montironi C, Leow WQ, Esteban-Fabro R, Pinyol R, Torres-Martin M, et al. Molecular classification and therapeutic targets in extrahepatic cholangiocarcinoma. J Hepatol 2020;73:315-327.

29. DePristo MA, Banks E, Poplin R, Garimella KV, Maguire JR, Hartl C, Philippakis AA, et al. A framework for variation discovery and genotyping using next-generation DNA sequencing data. Nat Genet 2011;43:491-498.

30. Mermel CH, Schumacher SE, Hill B, Meyerson ML, Beroukhim R, Getz G. GISTIC2.0 facilitates sensitive and confident localization of the targets of focal somatic copy-number alteration in human cancers. Genome Biol 2011;12:R41.

31. Gao Q, Zhu H, Dong L, Shi W, Chen R, Song Z, Huang C, et al. Integrated Proteogenomic Characterization of HBV-Related Hepatocellular Carcinoma. Cell 2019;179:561-577 e522.

32. Diaz-Gay M, Vila-Casadesus M, Franch-Exposito S, Hernandez-Illan E, Lozano JJ, Castellvi-Bel S. Mutational Signatures in Cancer (MuSiCa): a web application to implement mutational signatures analysis in cancer samples. BMC Bioinformatics 2018;19:224.

33. Wang S, Tao Z, Wu T, Liu XS. Sigflow: an automated and comprehensive pipeline for cancer genome mutational signature analysis. Bioinformatics 2021;37:1590-1592.

34. Chalmers ZR, Connelly CF, Fabrizio D, Gay L, Ali SM, Ennis R, Schrock A, et al. Analysis of 100,000 human cancer genomes reveals the landscape of tumor mutational burden. Genome Med 2017;9:34.

35. Alvarez MJ, Shen Y, Giorgi FM, Lachmann A, Ding BB, Ye BH, Califano A. Functional characterization of somatic mutations in cancer using network-based inference of protein activity. Nat Genet 2016;48:838-847.

36. Subramanian A, Tamayo P, Mootha VK, Mukherjee S, Ebert BL, Gillette MA, Paulovich A, et al. Gene set enrichment analysis: a knowledge-based approach for interpreting genome-wide expression profiles. Proc Natl Acad Sci U S A 2005;102:15545-15550.

37. Garcia-Alonso L, Holland CH, Ibrahim MM, Turei D, Saez-Rodriguez J. Benchmark and integration of resources for the estimation of human transcription factor activities. Genome Res 2019;29:1363-1375.

38. Uhrig S, Ellermann J, Walther T, Burkhardt P, Frohlich M, Hutter B, Toprak UH, et al. Accurate and efficient detection of gene fusions from RNA sequencing data. Genome Res 2021;31:448-460.

39. Kotliar D, Veres A, Nagy MA, Tabrizi S, Hodis E, Melton DA, Sabeti PC. Identifying gene expression programs of cell-type identity and cellular activity with single-cell RNA-Seq. Elife 2019;8.

40. Monti S, Tamayo P, Mesirov J, Golub T. Consensus clustering: A resampling-based method for class discovery and visualization of gene expression microarray data. Machine Learning 2003;52:91-118.

41. Wilkerson MD, Hayes DN. ConsensusClusterPlus: a class discovery tool with confidence assessments and item tracking. Bioinformatics 2010;26:1572-1573.

42. Casado P, Rodriguez-Prados JC, Cosulich SC, Guichard S, Vanhaesebroeck B, Joel S, Cutillas PR. Kinase-substrate enrichment analysis provides insights into the heterogeneity of signaling pathway activation in leukemia cells. Sci Signal 2013;6:rs6.

43. Wiredja DD, Koyuturk M, Chance MR. The KSEA App: a web-based tool for kinase activity inference from quantitative phosphoproteomics. Bioinformatics 2017;33:3489-3491.

44. Hornbeck PV, Chabra I, Kornhauser JM, Skrzypek E, Zhang B. PhosphoSite: A bioinformatics resource dedicated to physiological protein phosphorylation. Proteomics 2004;4:1551-1561.

45. Diella F, Gould CM, Chica C, Via A, Gibson TJ. Phospho.ELM: a database of phosphorylation sites--update 2008. Nucleic Acids Res 2008;36:D240-244.

46. Yang CY, Chang CH, Yu YL, Lin TC, Lee SA, Yen CC, Yang JM, et al. PhosphoPOINT: a comprehensive human kinase interactome and phospho-protein database. Bioinformatics 2008;24:i14-20.

47. Schwartz D, Gygi SP. An iterative statistical approach to the identification of protein phosphorylation motifs from large-scale data sets. Nat Biotechnol 2005;23:1391-1398.

48. Krug K, Mertins P, Zhang B, Hornbeck P, Raju R, Ahmad R, Szucs M, et al. A Curated Resource for Phosphosite-specific Signature Analysis. Mol Cell Proteomics 2019;18:576-593.

49. Linding R, Jensen LJ, Ostheimer GJ, van Vugt MA, Jorgensen C, Miron IM, Diella F, et al. Systematic discovery of in vivo phosphorylation networks. Cell 2007;129:1415-1426.

50. Cotto KC, Wagner AH, Feng YY, Kiwala S, Coffman AC, Spies G, Wollam A, et al. DGIdb 3.0: a redesign and expansion of the drug-gene interaction database. Nucleic Acids Res 2018;46:D1068-D1073.

51. Uhlen M, Bjorling E, Agaton C, Szigyarto CA, Amini B, Andersen E, Andersson AC, et al. A human protein atlas for normal and cancer tissues based on antibody proteomics. Mol Cell Proteomics 2005;4:1920-1932.

52. Pedregosa F, Varoquaux G, Gramfort A, Michel V, Thirion B, Grisel O, Blondel M, et al. Scikit-learn: Machine Learning in Python. ArXiv 2011;abs/1201.0490.

53. Wu T, Hu E, Xu S, Chen M, Guo P, Dai Z, Feng T, et al. clusterProfiler 4.0: A universal enrichment tool for interpreting omics data. The Innovation 2021;2:100141.

54. Shannon P, Markiel A, Ozier O, Baliga NS, Wang JT, Ramage D, Amin N, et al. Cytoscape: a software environment for integrated models of biomolecular interaction networks. Genome Res 2003;13:2498-2504.

55. Ellis MJ, Suman VJ, Hoog J, Goncalves R, Sanati S, Creighton CJ, DeSchryver K, et al. Ki67 Proliferation Index as a Tool for Chemotherapy Decisions During and After Neoadjuvant Aromatase Inhibitor Treatment of Breast Cancer: Results From the American College of Surgeons Oncology Group Z1031 Trial (Alliance). J Clin Oncol 2017;35:1061-1069.

56. Whitfield ML, Sherlock G, Saldanha AJ, Murray JI, Ball CA, Alexander KE, Matese JC, et al. Identification of genes periodically expressed in the human cell cycle and their expression in tumors. Mol Biol Cell 2002;13:1977-2000.

57. Aran D, Hu Z, Butte AJ. xCell: digitally portraying the tissue cellular heterogeneity landscape. Genome Biol 2017;18:220.

58. Yoshihara K, Shahmoradgoli M, Martinez E, Vegesna R, Kim H, Torres-Garcia W, Trevino V, et al. Inferring tumour purity and stromal and immune cell admixture from expression data. Nat Commun 2013;4:2612.

59. Hanzelmann S, Castelo R, Guinney J. GSVA: gene set variation analysis for microarray and RNA-seq data. BMC Bioinformatics 2013;14:7.

60. Li F, He X, Ye D, Lin Y, Yu H, Yao C, Huang L, et al. NADP(+)-IDH Mutations Promote Hypersuccinylation that Impairs Mitochondria Respiration and Induces Apoptosis Resistance. Mol Cell 2015;60:661-675.

61. Mei X, Qi D, Zhang T, Zhao Y, Jin L, Hou J, Wang J, et al. Inhibiting MARSs reduces hyperhomocysteinemia-associated neural tube and congenital heart defects. EMBO Mol Med 2020;12:e9469.

62. Li Y, Yao CF, Xu FJ, Qu YY, Li JT, Lin Y, Cao ZL, et al. APC/C(CDH1) synchronizes ribose-5-phosphate levels and DNA synthesis to cell cycle progression. Nat Commun 2019;10:2502.

63. Ghemrawi R, Pooya S, Lorentz S, Gauchotte G, Arnold C, Gueant JL, Battaglia-Hsu SF. Decreased vitamin B12 availability induces ER stress through impaired SIRT1-deacetylation of HSF1. Cell Death Dis 2013;4:e553.

64. Greco T, Vespa PM, Prins ML. Alternative substrate metabolism depends on cerebral metabolic state following traumatic brain injury. Exp Neurol 2020;329:113289.

65. Jones AJ, Hirst J. A spectrophotometric coupled enzyme assay to measure the activity of succinate dehydrogenase. Anal Biochem 2013;442:19-23.

66. Blanco-Dominguez R, Sanchez-Diaz R, de la Fuente H, Jimenez-Borreguero LJ, Matesanz-Marin A, Relano M, Jimenez-Alejandre R, et al. A Novel Circulating MicroRNA for the Detection of Acute Myocarditis. N Engl J Med 2021;384:2014-2027.
