# Supplementary material for: Proteogenomic characterization of cholangiocarcinoma
Source: Hepatology. 2022 Jul 5;77(2):411–29. doi: 10.1002/hep.32624 (PMC9869950; doi:10.1002/hep.32624)

Supporting Figure 2

A

Protein: CDK1 Sequence: VYTHEVVTLWYR, T161-Phos (79.96633 Da)

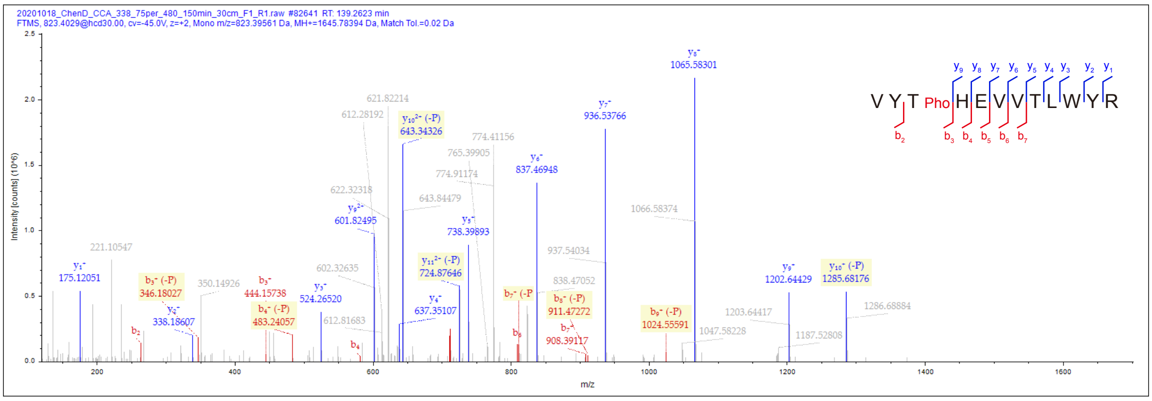

Protein: MCM2 Sequence: GNDPLTSSPGR, S27-Phos (79.96633 Da)

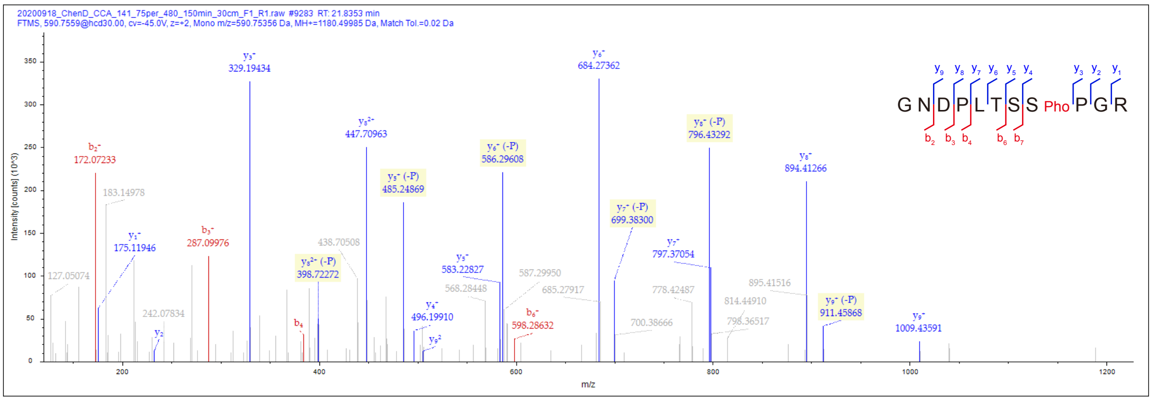

Protein: RB1 Sequence: KSNLDEEVNVIPPHTPVR, T373-Phos (79.96633 Da)

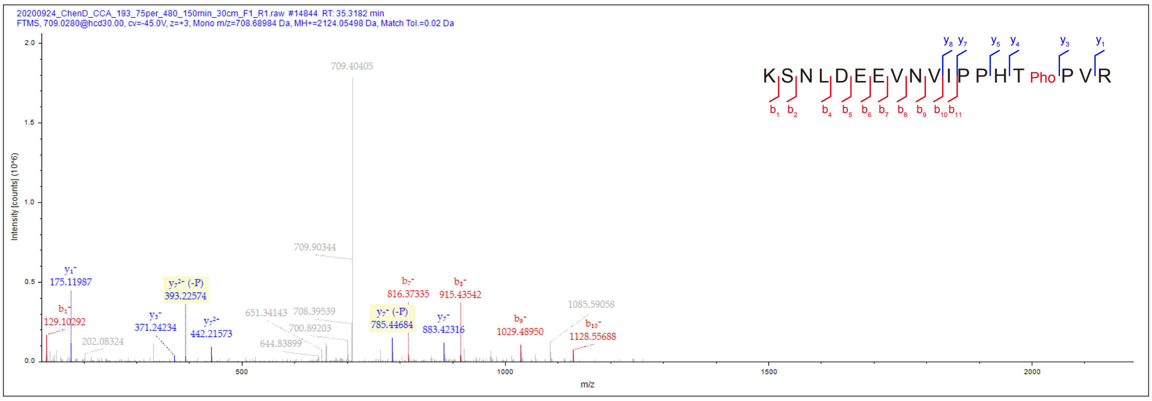

B

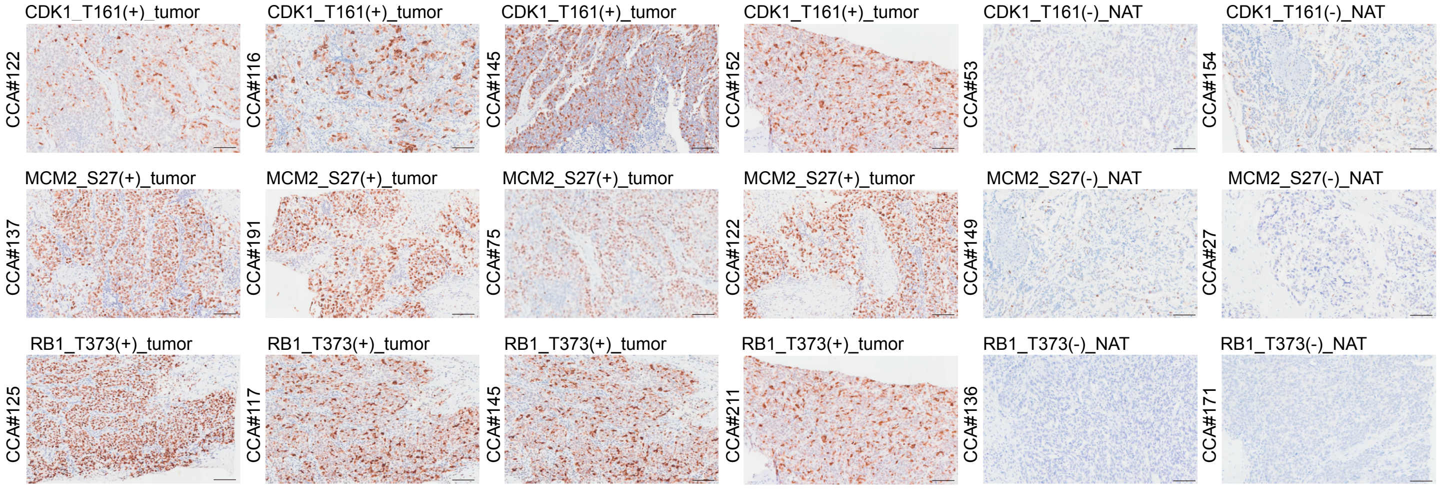

Supplement: Supplementary file 2 [file hep-77-411-s002.pdf]
