# Supplementary figures and images for: Proteogenomic characterization of cholangiocarcinoma
Source: Hepatology. 2022 Jul 5;77(2):411–29. doi: 10.1002/hep.32624 (PMC9869950; doi:10.1002/hep.32624)

# Supporting Figure 3

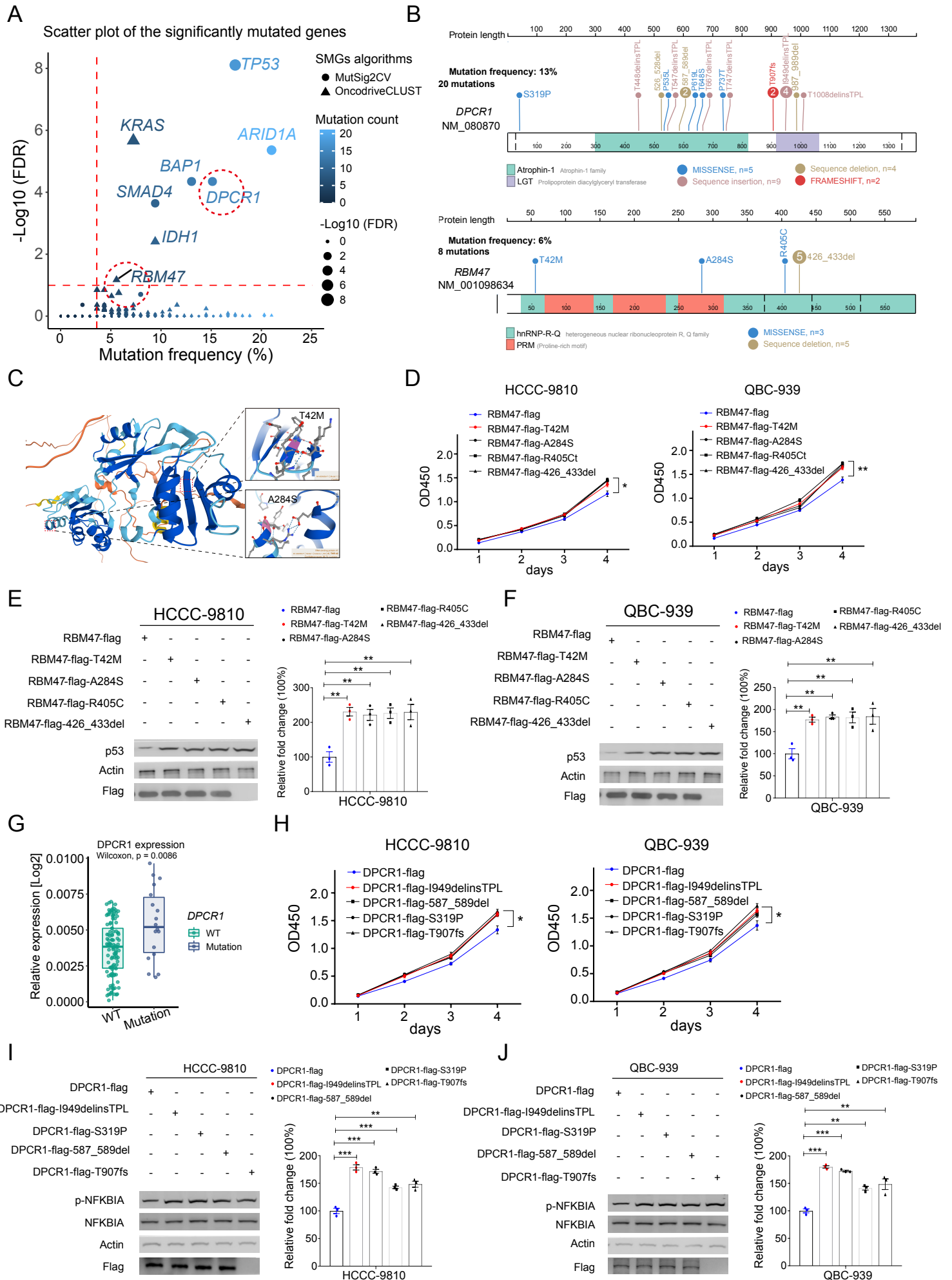

Supplement: Supplementary file 3 [file hep-77-411-s003.pdf]

# Supporting Figure 4

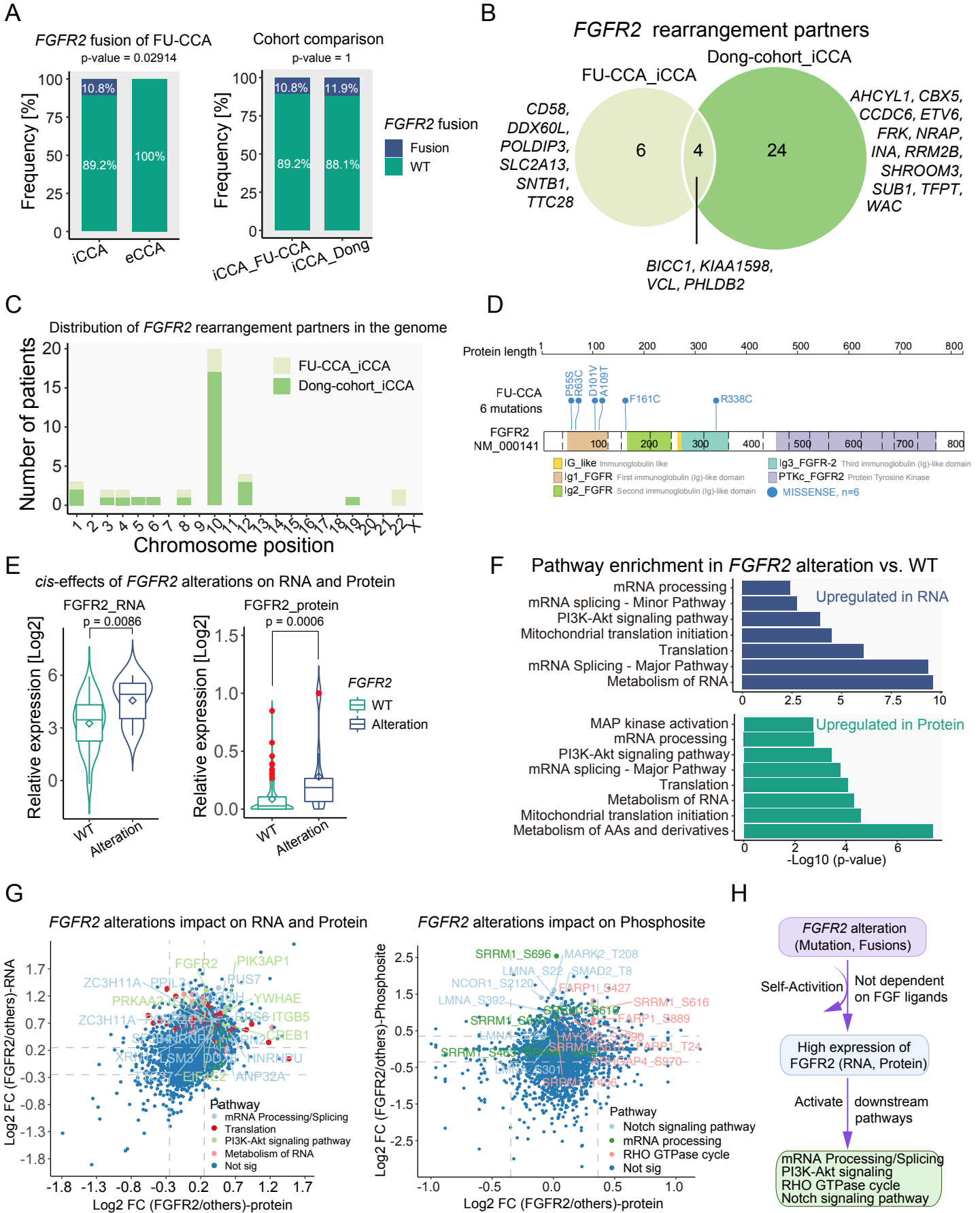

Supplement: Supplementary file 4 [file hep-77-411-s004.pdf]

# Supporting Figure 5

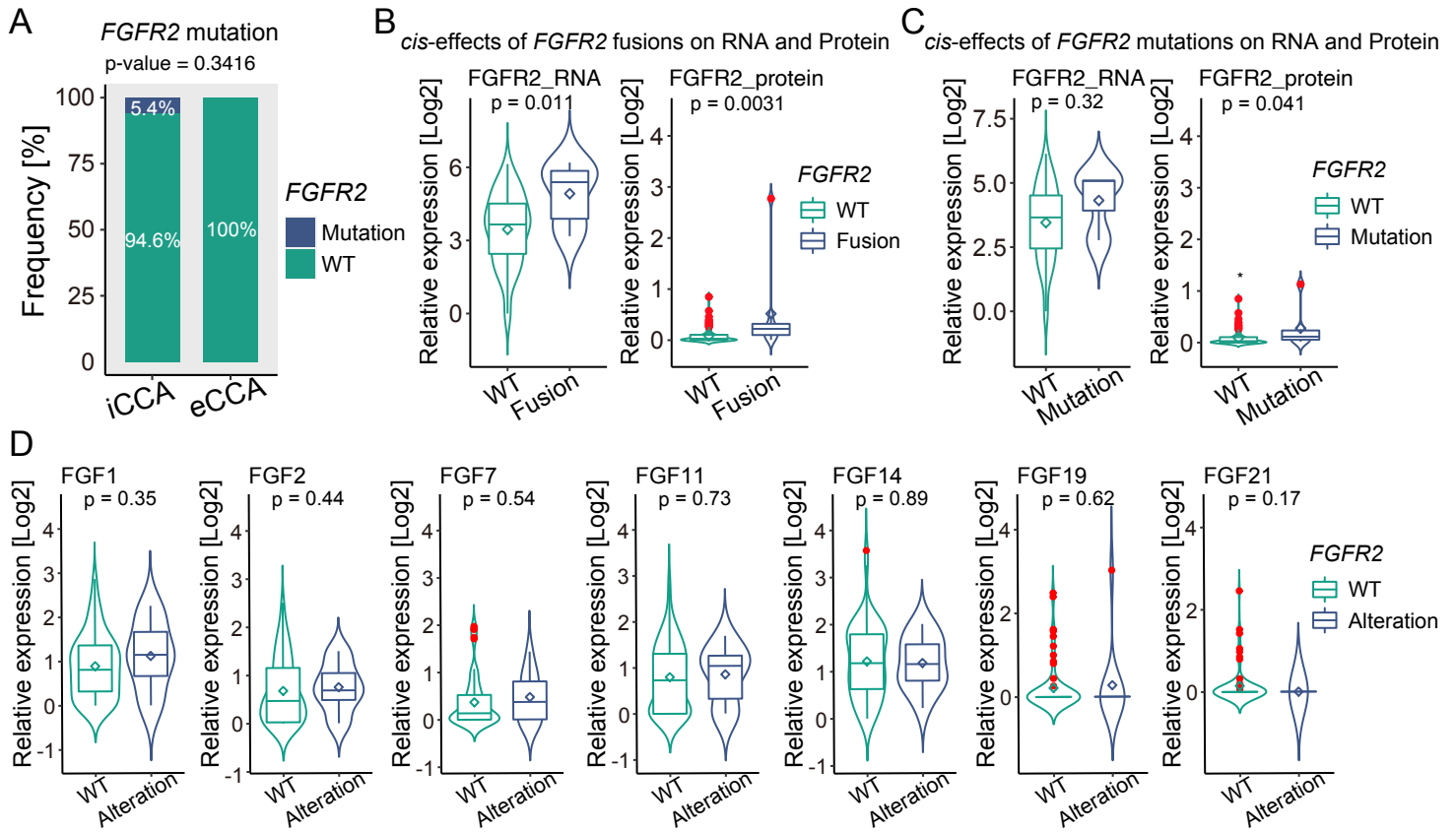

Supplement: Supplementary file 5 [file hep-77-411-s005.pdf]

# Supporting Figure 6

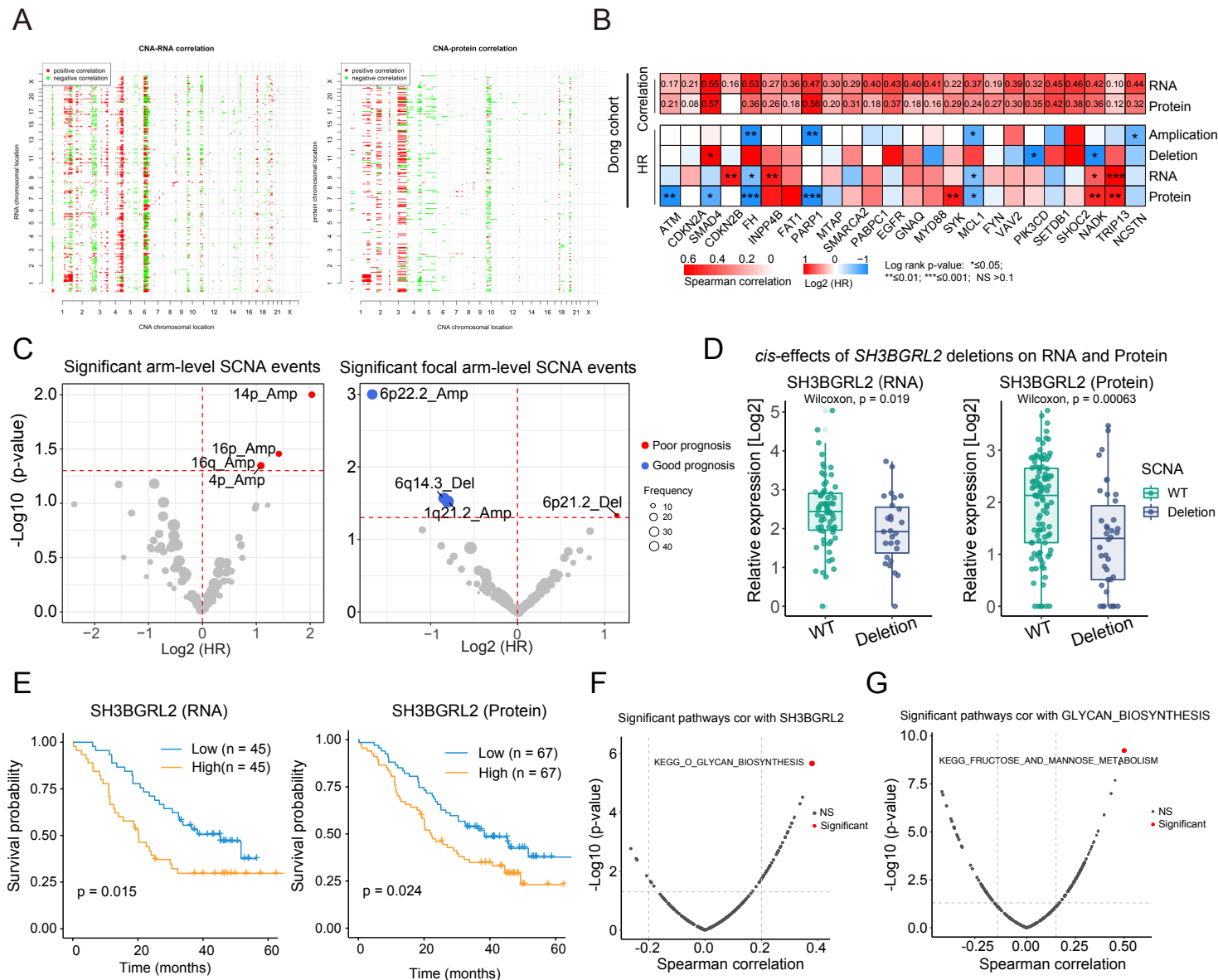

Supplement: Supplementary file 6 [file hep-77-411-s006.pdf]

# Supporting Figure 8

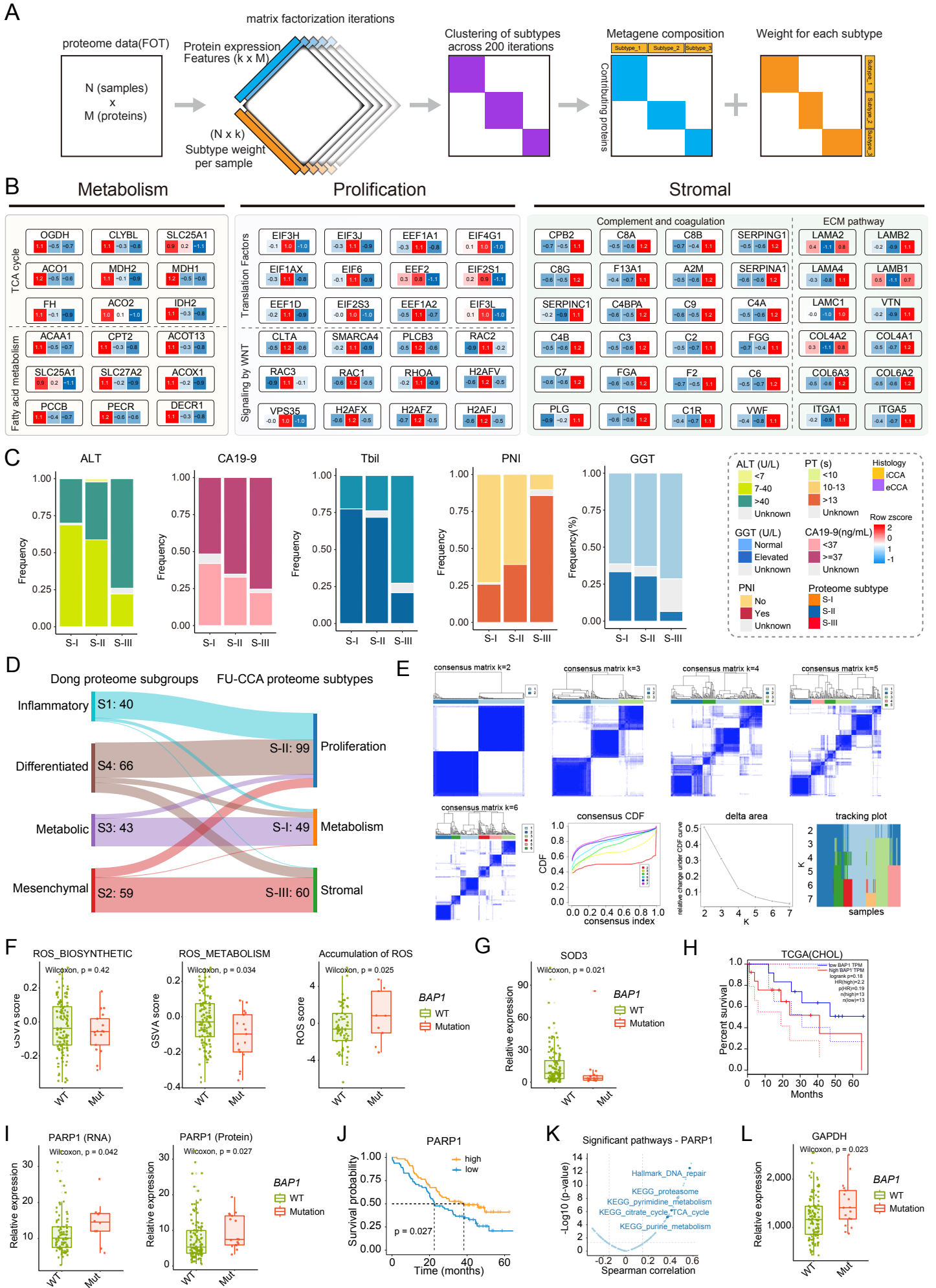

Supplement: Supplementary file 7 [file hep-77-411-s007.pdf]

# Supporting Figure 9

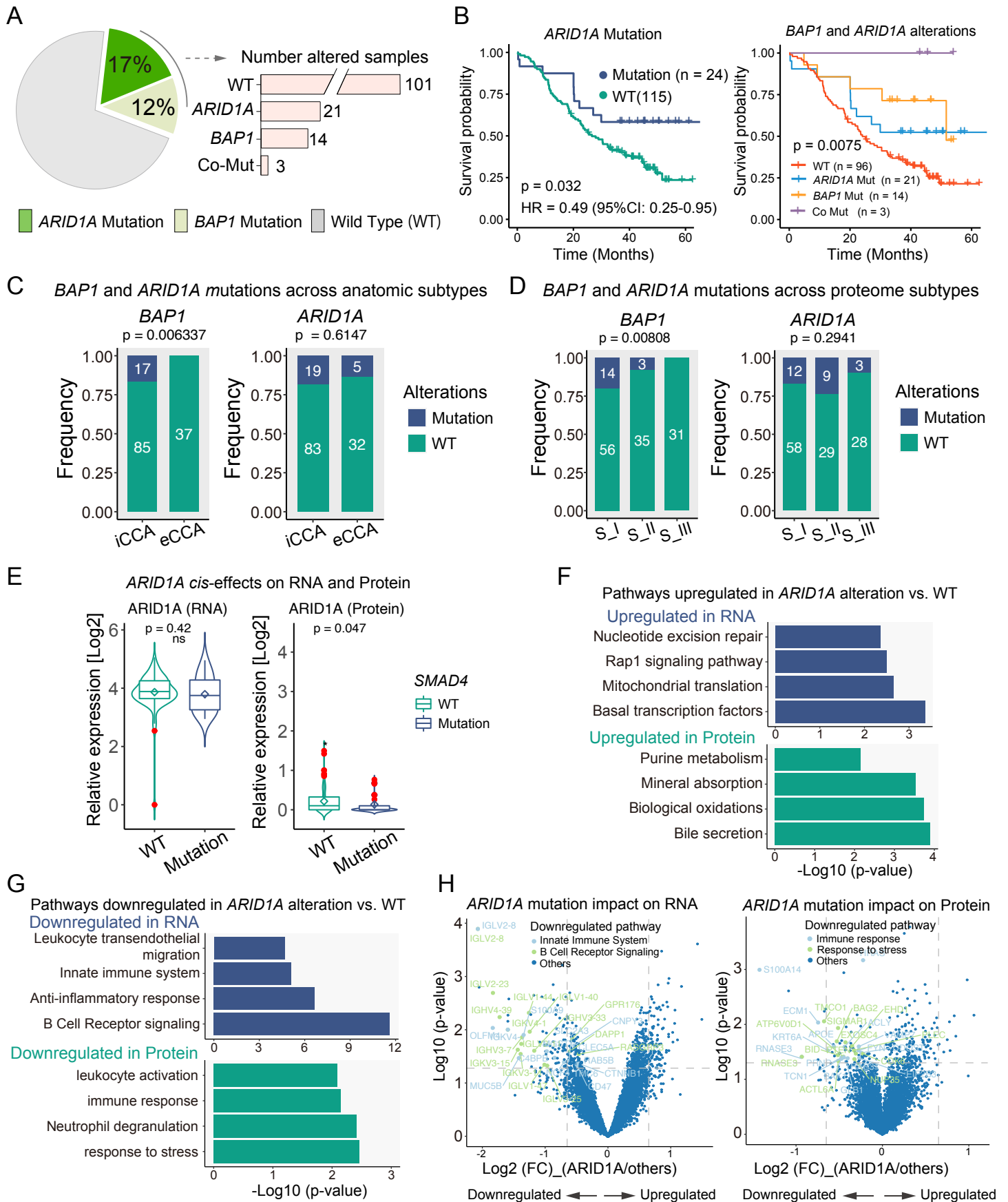

Supplement: Supplementary file 8 [file hep-77-411-s008.pdf]

# Supporting Figure 10

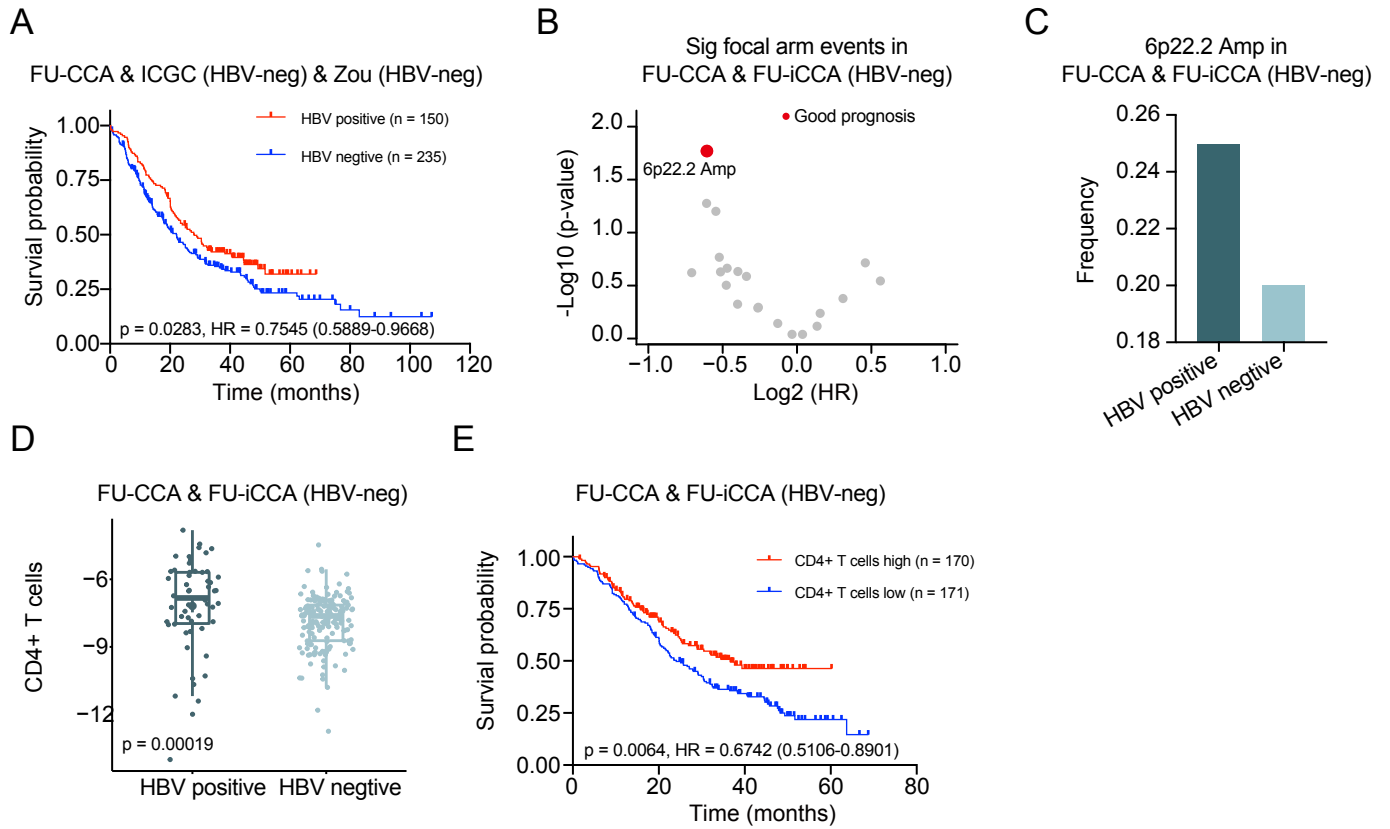

Supplement: Supplementary file 9 [file hep-77-411-s009.pdf]

# Supporting Figure 11

B

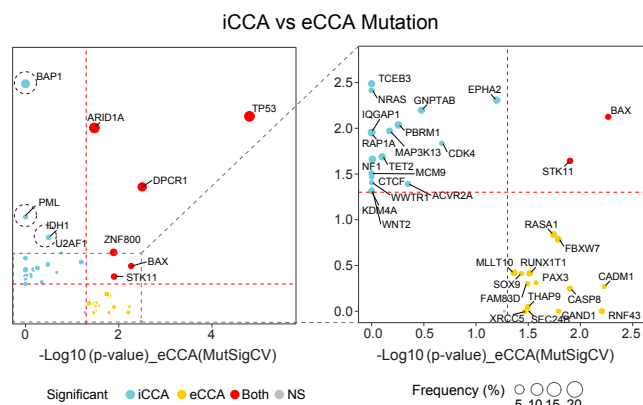

F

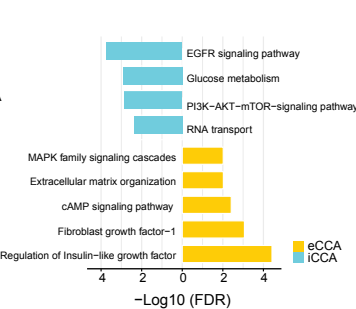

J

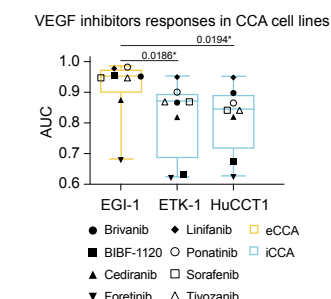

M

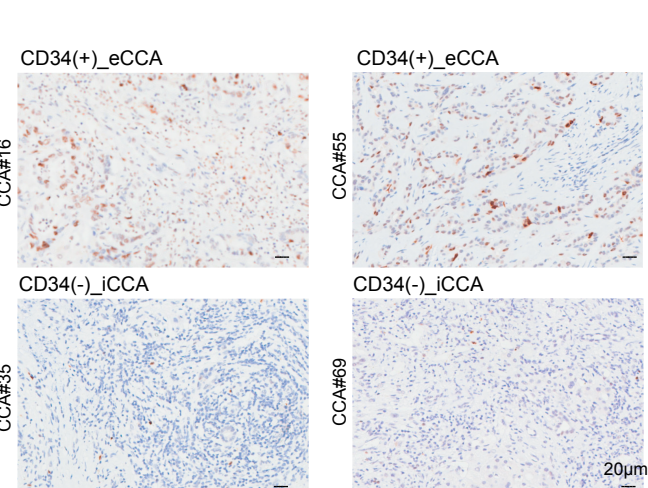

Supplement: Supplementary file 10 [file hep-77-411-s010.pdf]

# Supporting Figure 12

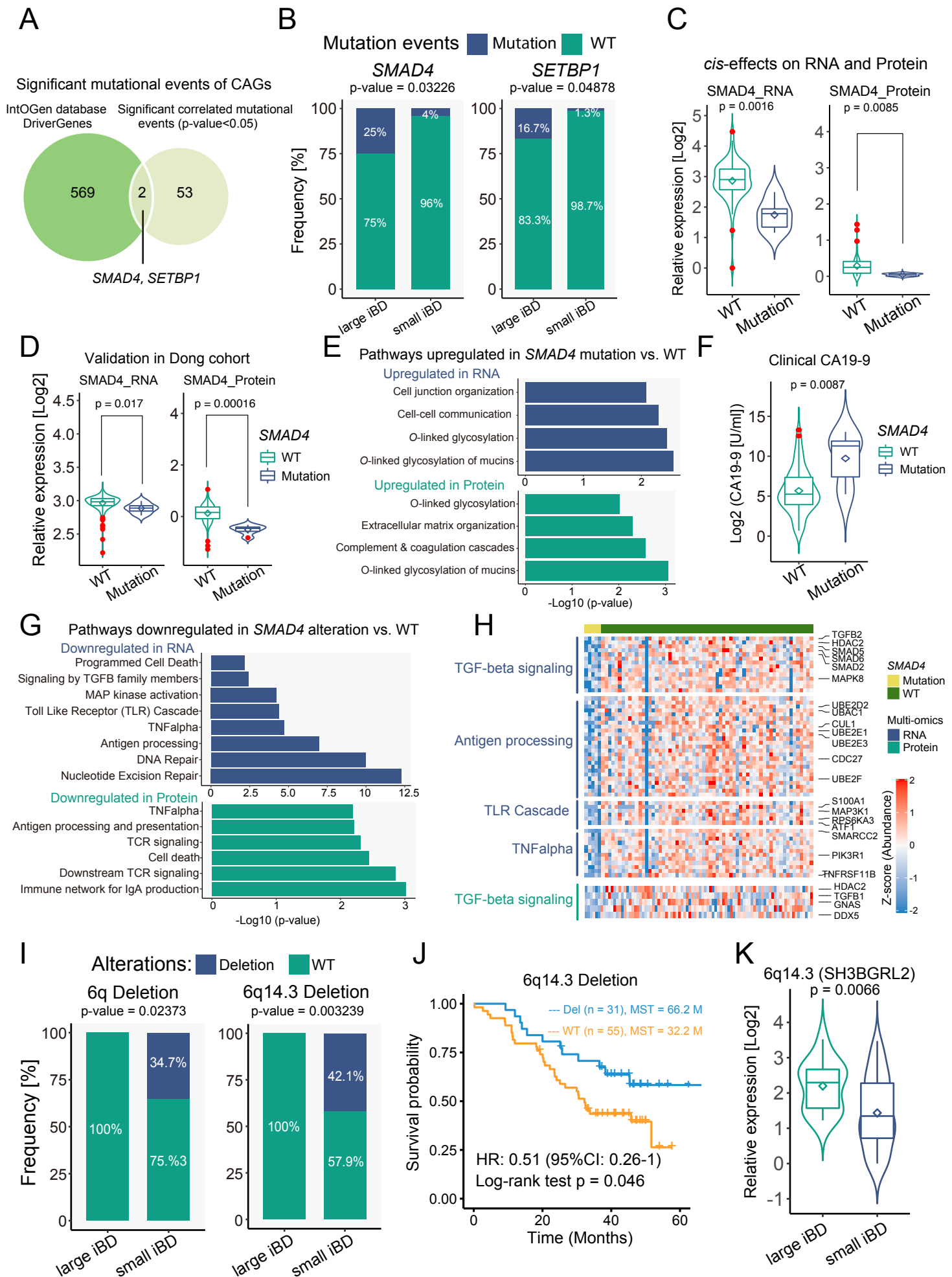

Supplement: Supplementary file 11 [file hep-77-411-s011.pdf]

## Supporting Figure 13

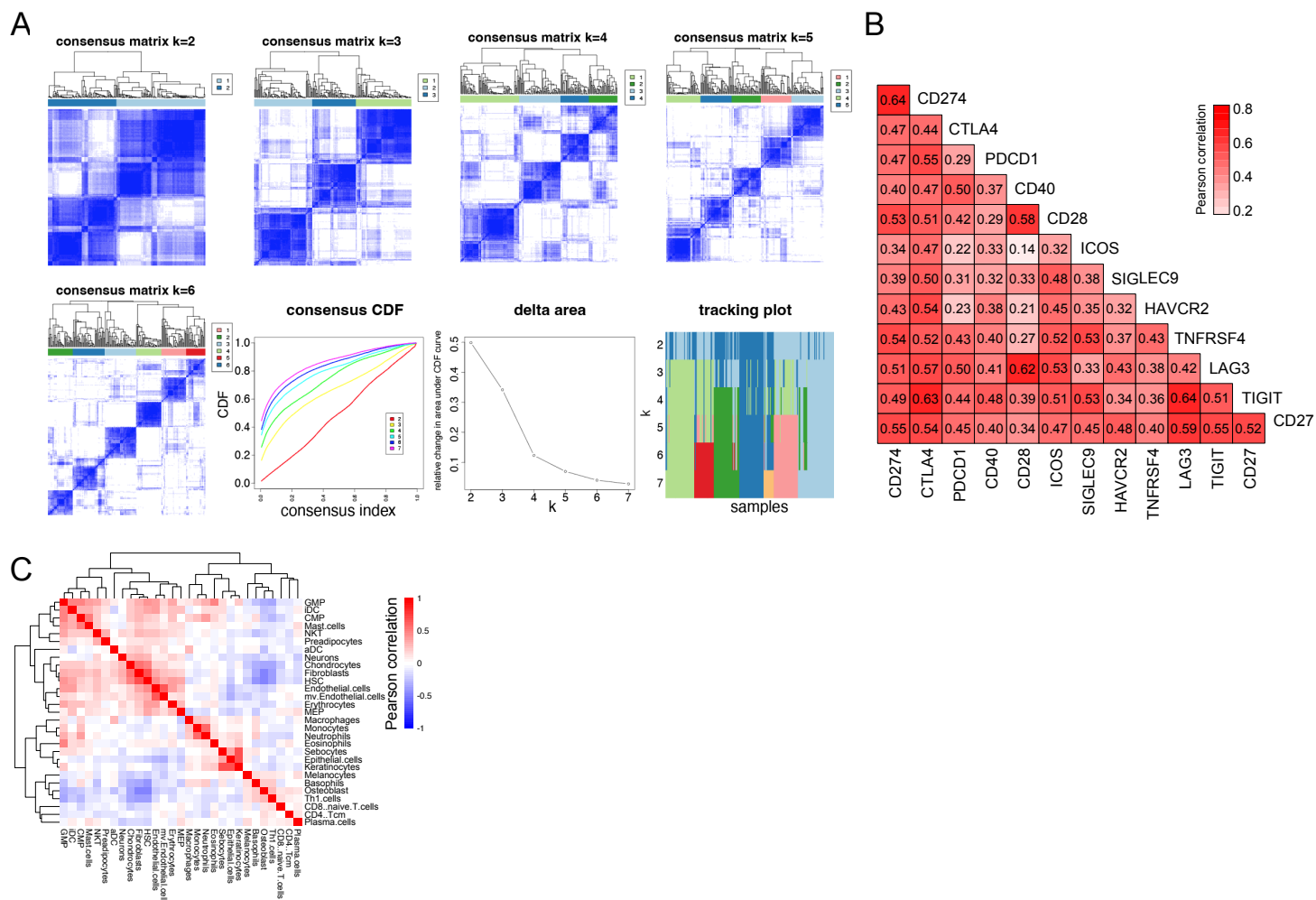

Supplement: Supplementary file 12 [file hep-77-411-s012.pdf]
